# Swimming Motility Mediates the Formation of Neutrophil Extracellular Traps Induced by Flagellated *Pseudomonas aeruginosa*

**DOI:** 10.1371/journal.ppat.1005987

**Published:** 2016-11-17

**Authors:** Madison Floyd, Matthew Winn, Christian Cullen, Payel Sil, Benoit Chassaing, Dae-goon Yoo, Andrew T. Gewirtz, Joanna B. Goldberg, Linda L. McCarter, Balázs Rada

**Affiliations:** 1 College of Veterinary Medicine, Department of Infectious Diseases, The University of Georgia, Athens, Georgia, United States of America; 2 Center for Inflammation, Immunity, & Infection, Institute for Biomedical Sciences, Georgia State University, Atlanta, Georgia, United States of America; 3 Division of Pulmonology, Allergy/Immunology, Cystic Fibrosis and Sleep, Department of Pediatrics, Emory University School of Medicine, Atlanta, Georgia, United States of America; 4 Carver College of Medicine, Department of Microbiology, The University of Iowa, Iowa City, Iowa, United States of America; University of Maryland, UNITED STATES

## Abstract

*Pseudomonas aeruginosa* is an opportunistic pathogen causing severe infections often characterized by robust neutrophilic infiltration. Neutrophils provide the first line of defense against *P*. *aeruginosa*. Aside from their defense conferred by phagocytic activity, neutrophils also release neutrophil extracellular traps (NETs) to immobilize bacteria. Although NET formation is an important antimicrobial process, the details of its mechanism are largely unknown. The identity of the main components of *P*. *aeruginosa* responsible for triggering NET formation is unclear. In this study, our focus was to identify the main bacterial factors mediating NET formation and to gain insight into the underlying mechanism. We found that *P*. *aeruginosa* in its exponential growth phase promoted strong NET formation in human neutrophils while its NET-inducing ability dramatically decreased at later stages of bacterial growth. We identified the flagellum as the primary component of *P*. *aeruginosa* responsible for inducing NET extrusion as flagellum-deficient bacteria remained seriously impaired in triggering NET formation. Purified *P*. *aeruginosa* flagellin, the monomeric component of the flagellum, does not stimulate NET formation in human neutrophils. *P*. *aeruginosa*-induced NET formation is independent of the flagellum-sensing receptors TLR5 and NLRC4 in both human and mouse neutrophils. Interestingly, we found that flagellar motility, not flagellum binding to neutrophils per se, mediates NET release induced by flagellated bacteria. Immotile, flagellar motor-deficient bacterial strains producing paralyzed flagella did not induce NET formation. Forced contact between immotile *P*. *aeruginosa* and neutrophils restored their NET-inducing ability. Both the *motAB* and *motCD* genetic loci encoding flagellar motor genes contribute to maximal NET release; however the *motCD* genes play a more important role. Phagocytosis of *P*. *aeruginosa* and superoxide production by neutrophils were also largely dependent upon a functional flagellum. Taken together, the flagellum is herein presented for the first time as the main organelle of planktonic bacteria responsible for mediating NET release. Furthermore, flagellar motility, rather than binding of the flagellum to flagellum-sensing receptors on host cells, is required for *P*. *aeruginosa* to induce NET release.

## Author Summary

*Pseudomonas aeruginosa* leaves a large footprint in human disease because it causes infections in immunocompromised patients. Its ability to quickly adapt to diverse environments and to form biofilms poses a significant challenge to the medical community. Neutrophil granulocytes, professional phagocytes found cruising through the body’s circulatory system and tissues, provide the most efficient immune response against *P*. *aeruginosa*. Neutrophils utilize multiple strategies to eliminate bacteria. Formation of neutrophil extracellular traps (NETs), a DNA-based scaffold with attached antimicrobial proteins, provides an efficient mechanism to trap *P*. *aeruginosa*. The detailed mechanism of NET release induced by bacteria remains unclear. Our data show that the flagellum, the organelle that provides swimming motility to *P*. *aeruginosa*, is the main factor required to induce NET release. Our novel findings indicate that the flagellum, and in particular swimming motility, mediates *P*. *aeruginosa*-induced NET extrusion independently of the well-characterized flagellin receptors. The novel data presented here also suggest that down-regulation of flagellar motility characteristically seen in *P*. *aeruginosa* lung infections in cystic fibrosis is relevant for *P*. *aeruginosa* to avoid neutrophil attacks.

## Introduction

*Pseudomonas aeruginosa* is a ubiquitous opportunistic Gram-negative pathogen found in the environment. *P*. *aeruginosa* rarely infects healthy individuals and mainly causes lung infections in patients with compromised immune defenses [cystic fibrosis (CF), chronic obstructive pulmonary disease (COPD), HIV, non-CF bronchiectasis and hospital-acquired pneumonia] [1–6]. *P*. *aeruginosa* colonizes up to 80% of CF patients, 4–15% of COPD patients, 8–25% of HIV patients with pneumonia, 28% of non-CF bronchiectasis patients and 18–20% of patients with hospital-acquired pneumonia [4, 7–9]. The high incidence of *P*. *aeruginosa* infections among these patients demonstrates that this bacterium represents a serious clinical problem.

Polymorphonuclear neutrophilic granulocytes (PMN) play a critical role in fighting *P*. *aeruginosa*. Mammalian species lacking phagocytic cells or innate immune defense molecules are highly susceptible to infection with *P*. *aeruginosa* [10–12]. Humans deficient in key neutrophil-mediated antimicrobial mechanisms, such as specific granule deficiency or leukocyte adhesion deficiency (LAD), are prone to *P*. *aeruginosa* infection [10]. Neutropenia, caused by chemotherapy, HIV infection or autoimmune disorders, predisposes patients to *P*. *aeruginosa* pneumonia [13–15]. Only patients with the full defensive arsenal of PMNs are able to defeat *P*. *aeruginosa* infections. An adequate immune response to *P*. *aeruginosa* requires the full spectrum of neutrophilic defenses.

PMNs are the first to arrive at the site of infection where they fight pathogens via various mechanisms. In addition to phagocytic killing [16], PMNs also trap and kill microbes via an alternative mechanism known as Neutrophil Extracellular Trap (NET) formation [17]. NETs are composed of a DNA scaffold associated with histones and neutrophil granule components, such as myeloperoxidase (MPO) and neutrophil elastase (NE) [17–19]. Only NET-forming PMNs and not apoptotic or necrotic PMNs release protein-DNA complexes (MPO-DNA, NE-DNA or histone-DNA) [17, 20–22]. Signaling pathways leading to NET formation are largely unknown. The few known players are: NADPH oxidase, MPO, HNE (human neutrophil elastase) and histone citrullination mediated by peptidylarginine deiminase 4 (PAD4) [23, 24]. Both MPO and HNE are required for NET release [23]. The neutrophil respiratory burst produced by the NADPH oxidase is also essential for induction of NET formation by most bacterial stimuli studied [25, 26]. PAD4-mediated citrullination of histones is required for NET formation [27, 28]. These citrullinated histones are only present in NETs, not in resting PMNs [29] and PAD4-deficient murine PMNs do not form NETs [28, 30]. PAD4-deficient mice have impaired NET-mediated antibacterial defenses [27].

Robust neutrophil infiltration and NETs have been detected in most of the diseases associated with *P*. *aeruginosa* lung infection [31–39]. This suggests that *P*. *aeruginosa-*triggered NET formation takes place *in vivo* under those disease conditions. Several independent studies performed by us and other groups confirmed that *P*. *aeruginosa* induces robust NET release in human PMNs [19, 33, 40–45]. We found that *P*. *aeruginosa*-induced NET formation requires the NADPH oxidase that leads to the release of citrullinated histones [19, 41]. However, the mechanism by which *P*. *aeruginosa* initiates NET extrusion from PMNs remains unknown.

Here, we aimed to identify components of planktonic bacteria and their associated mechanism(s) responsible for inducing NET release in PMNs. We identified the flagellum as the main bacterial component required to trigger maximal NET release. Interestingly, flagellum-mediated swimming motility, and not flagellum production itself, proved to be the main inciting mechanism. Our studies provide novel insight into *P*. *aeruginosa*-induced NET formation, a host-microbe interaction clinically relevant in several airway diseases.

## Results

### The extent of NET formation induced by *P*. *aeruginosa* depends on the bacterial growth phase

**Fig 1:**
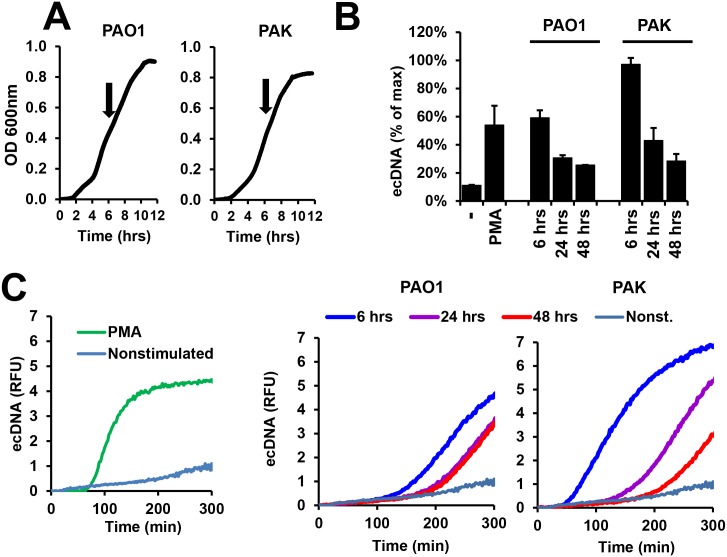
Planktonic *P*. *aeruginosa* in its exponential growth phase induces the most robust NET release in human PMNs. **(A)** Characteristic growth curves of *P*. *aeruginosa* PAO1 and PAK strains in LB liquid medium (shaken cultures) recorded as optical density measured at 600 nm for 12 hrs. Arrows indicate bacterial densities at 6-hr incubation times. Mean of *n* = 4 experiments. **(B)** *P*. *aeruginosa* PAK and PAO1 strains were cultured for the indicated times, washed and exposed to human PMNs for 5 hrs (10 MOI) for measurement of extracellular DNA release by Sytox Orange fluorescence. Mean+/-S.E.M., *n* = 2. **(C)** Representative kinetic curves of DNA release (Sytox Orange fluorescence) of the endpoint data presented in panel B. The left graph shows non-stimulated human PMNs and those activated by 100 nM PMA. The middle graph shows curves after PAK stimulation. The right graph presents data following PAO1 exposure. PMA, phorbol myristate acetate. *n* = 2.

Although bacteria have been shown to trigger NET release, it is unknown which microbial components mediate this process. To gain insight into this question, we monitored *P*. *aeruginosa*’s ability to trigger NET release at various phases of growth as *P*. *aeruginosa* expresses different phenotypic features depending on its growth phase [46]. Early exponential phase cultures are characterized by motility and expression of virulence factors, while in later growth phases these features are lost and quorum-sensing molecules and extracellular polysaccharides become expressed to a greater extent [47]. We used early exponential phase (OD = 0.4 at 600nm, ~6hrs incubation) (Fig 1A), early stationary phase (~24 hrs) and late stationary phase (~48 hrs) cultures of two laboratory strains of *P*. *aeruginosa*, type A flagellin-producing PAK and type B flagellin-producing PAO1 [48]. PMA (phorbol 12-myristate 13-acetate, a potent activator of PKC) is capable of inducing robust NET release by PMNs [19] and was utilized as a positive control. At the indicated times, bacteria were washed and exposed to human PMNs to measure NET release. As Fig 1B and 1C show, early exponential phase cultures of both strains induced the greatest NET release with decreasing induction of NET release by bacteria at each subsequent time. The PAK strain reproducibly induced larger amounts of NETs than PAO1 (Fig 1B and 1C). These data show that *P*. *aeruginosa* in its early exponential growth phase triggers the most robust NET release.

### Characterization of flagellum-deficient *P*. *aeruginosa* strains

This finding suggested that bacterial components expressed at this early growth stage but lost at later stages are the main inducers of NET release. Flagellum-promoted swimming motility is often a hallmark characteristic of planktonic bacteria in their exponential growth phase [49, 50]. To study the role of the flagellum in NET formation we used flagellum-deficient PAO1 and PAK strains (PAO1 *fliC* and PAK *flgC*). The *fliC* gene encodes the flagellin monomer that polymerizes to form the flagellar filament, and the *flgC* gene encodes the flagellar hook to which the flagellar filament attaches [51]. As expected, both flagellum-deficient strains were immotile whereas their parental, flagellated counterparts displayed strong swimming motility (S1 Fig).

### Establishing an ELISA assay quantitating *P*. *aeruginosa* flagellin levels

There are currently no commercially available methods to quantitate flagellin production in *P*. *aeruginosa*. Immunoblotting performed on bacterial lysates using an anti-*P*. *aeruginosa* flagellin antibody is described in the literature, but this method provides only semi-quantitative results [52]. Therefore, we developed an ELISA assay using a commercially available antibody capable of accurate quantitation of *P*. *aeruginosa* flagellin levels in bacterial lysates. Briefly, bacterial lysates are immobilized to the bottom of high-binding ELISA plates, blocked and exposed to anti-*P*. *aeruginosa* flagellin antibody, followed by repeated washes and addition of a secondary, peroxidase-labeled, anti-murine IgG antibody (S2A Fig). Reliable and highly reproducible standard curves can be established using commercially available, purified *P*. *aeruginosa* flagellin resulting in a tight correlation between flagellin levels and optical density (S2B Fig). To show the specificity of the assay for flagellin obtained from *P*. *aeruginosa*, we tested the ELISA assay with identical concentrations of *P*. *aeruginosa* and *Shigella flexneri* flagellin. The assay detected flagellin derived only from *P*. *aeruginosa*, not from *S*. *flexneri* (S2C Fig). With this new tool, we observed no flagellin expression by the PAK *flgC* strain, as opposed to confirmed flagellin expression by its parental strain (PAK WT) (S2D Fig).

### The flagellum is the main contributor to total MPO and HNE release induced by *P*. *aeruginosa*

**Fig 2:**
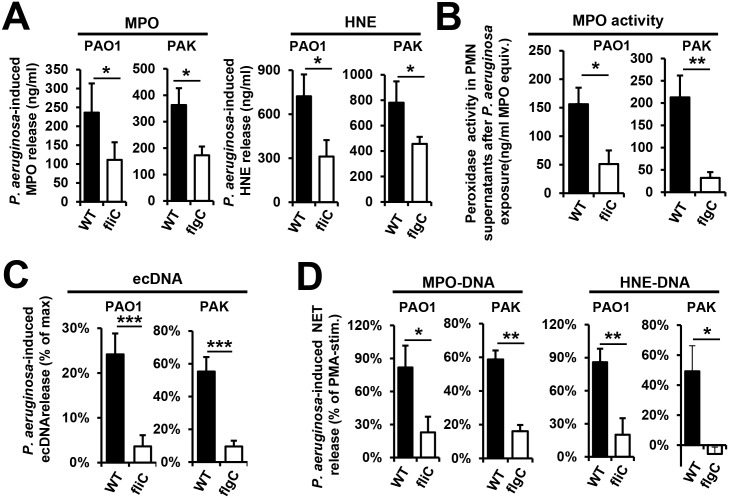
Flagellum is the main contributor to NET release triggered by *P*. *aeruginosa*. **(A)** Human PMNs were exposed to WT (PAK or PAO1) and flagellum-deficient strains (PAK *flgC* and PAO1 *fliC*) of *P*. *aeruginosa* (10 MOI), and the following readouts were measured: **(A)** Release of myeloperoxidase (MPO) and human neutrophil elastase (HNE) measured by ELISA. MPO: PAK (*n* = 5), PAO1 (*n* = 4); HNE: PAK (*n* = 11), PAO1 (*n* = 7). Values without bacterial stimulation were subtracted. **(B)** Enzymatic activity of MPO: PAK (*n* = 7), PAO1 (*n* = 7). Values without bacterial stimulation were subtracted. **(C)** DNA release (Sytox Orange fluorescence): PAO1 (*n* = 13), PAK (*n* = 3). **(D)** NET release measured by ELISA. MPO-DNA: PAK (*n* = 3), PAO1 (*n* = 5); HNE-DNA: PAK (*n* = 3), PAO1 (*n* = 5). Mean+/-S.E.M. Values of unstimulated PMNs were subtracted. *, p<0.05; **, p<0.01.

Previously, we demonstrated that human PMNs release active MPO and HNE in the presence of *P*. *aeruginosa* PA14 [19]. Fig 2A shows that PAO1 and PAK strains also induce MPO and HNE release in human PMNs. Flagellum-deficiency significantly reduced *P*. *aeruginosa*-triggered MPO release [PAO1: 53.5+/-12.3% reduction (*p* = 0.0495), PAK: 44.3+/-13.1% reduction (*p* = 0.0296)] (Fig 2A). HNE release was also reduced in the case of both strains [PAO1: 56.4+/-11.5% reduction (*p* = 0.0482), PAK: 32.0+/-6.0% (*p* = 0.0467)] (Fig 2A). Our previous data also show that MPO remains enzymatically active after being released from PA14-exposed PMNs [19]. This was also true using both PAO1 and PAK strains (Fig 2B). Flagellum-deficient strains induced significantly less release of active MPO than their corresponding wild-type strains [PAO1: 68.0+/-11.8% reduction (*p* = 0.020), PAK: 80.9+/-6.2% reduction (*p* = 0.004)] (Fig 2B). Thus, the bacterial flagellum is required to maximal release of HNE and active MPO from PMNs upon *P*. *aeruginosa* exposure, allowing for a more impactful immune response.

### Bacterial flagellum mediates *P*. *aeruginosa*-induced NET release

Our previously published data suggest that NET formation provides the primary mechanism of MPO and HNE release from PMNs in the presence of *P*. *aeruginosa* [19]. We next tested how flagellum deficiency affects *P*. *aeruginosa*-initiated NET release. Non-flagellated *P*. *aeruginosa* strains induced only minimal extracellular DNA release (ecDNA) while their flagellated counterparts triggered a signal closer to that induced by PMA in human PMNs (Fig 2C). Lack of flagellum resulted in a 74.1+/-6.3% (PAO1, *n* = 12) or 81.8+/-3.6% (PAK, mean+/-S.E.M., *n* = 5) reduction in ecDNA release (Fig 2C). To specifically quantitate NETs, we used established ELISA assays detecting NET-specific MPO-DNA and HNE-DNA complexes developed in our laboratory [41, 53]. These assays do not detect NET components alone (DNA, MPO, HNE or nucleosomes) (S3 Fig). We observed robust NET release triggered by the wild-type flagellated *P*. *aeruginosa* (PAO1 and PAK) but not by isogenic non-flagellated bacteria (Fig 2D). Lack of flagellum resulted in a reduction in *P*. *aeruginosa*-induced MPO-DNA release of 77.2+/-20.7% (PAK, *n* = 3) and 61.4+/-17.1% (PAO1, *n* = 3), as well as, a HNE-DNA release reduction of 88.0+/-12.5% (PAO1, *n* = 3) and 109.9+/-11.5% (PAK, mean+/-S.E.M., *n* = 3) (Fig 2D).

**Fig 3:**
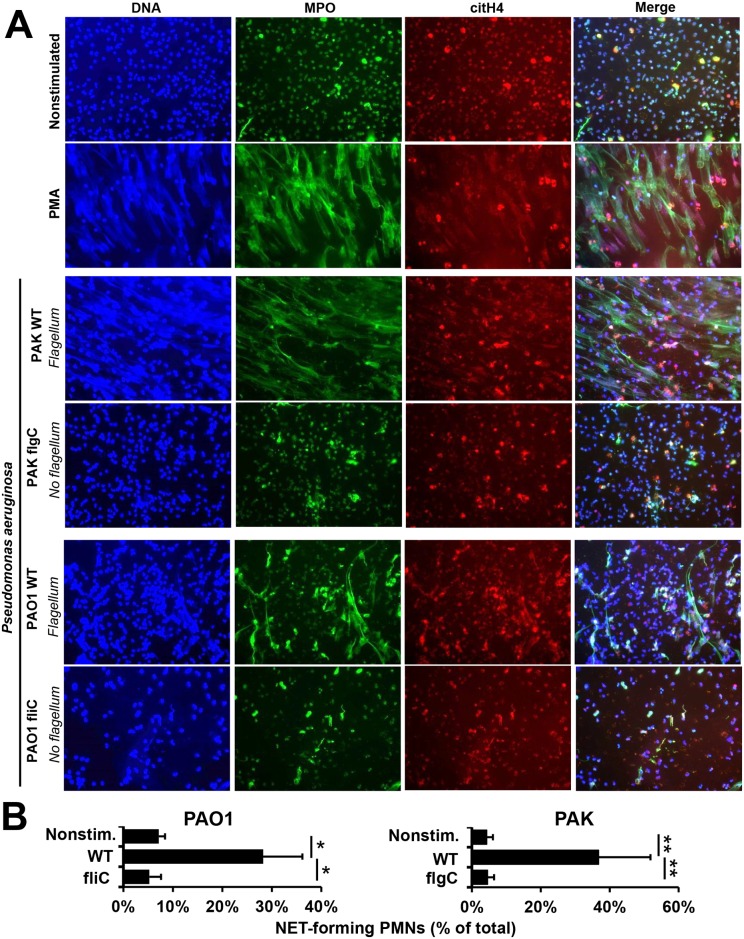
Immunofluorescence staining reveals that flagellum-deficiency abolishes *P*. *aeruginosa*-induced NET formation. Human PMNs were exposed to wild-type (WT) or flagellum-deficient strains of *P*. *aeruginosa* PAK *flgC* and PAO1 *fliC*, and NET release was documented. **(A)** Immunofluorescence staining of myeloperoxidase (MPO, green), citrullinated histone H4 (citH4, red), extracellular DNA (DAPI, blue) and their merged images are shown. Representative results, *n* = 3. **(B)** Quantitation of immunofluorescence images shown in panel (A). Based on nuclear morphology and co-staining with MPO and citH4, NET-forming PMNs were identified and their numbers quantitated compared to the total cell population (expressed as percentage of total). Mean+/-S.E.M., n = 3. One-way ANOVA, Tukey’s post hoc test. *, p<0.05; **, p<0.01. Nonstim., nonstimulated; WT, wild-type.

Since NETs have a distinctive morphology [17] and we had previously shown that MPO and citrullinated histone H4 co-localize with DNA in *P*. *aeruginosa*-induced NETs [19, 41], we compared immunofluorescence staining of human neutrophils exposed to flagellum-deficient bacteria to those exposed to wild-type strains. The absence of flagellum greatly reduced NET release, as assessed by the amount of characteristic DNA structures expelled from PMNs (Fig 3A and 3B). MPO and citrullinated histones co-localized with DNA in NETs triggered by flagellated *P*. *aeruginosa* (Fig 3A and 3B), further confirming that NET formation is the main neutrophil mechanism responding to *P*. *aeruginosa* [19, 41].

### Flagellum-deficiency impairs *P*. *aeruginosa*-induced phagocytosis and superoxide production in PMNs

**Fig 4:**
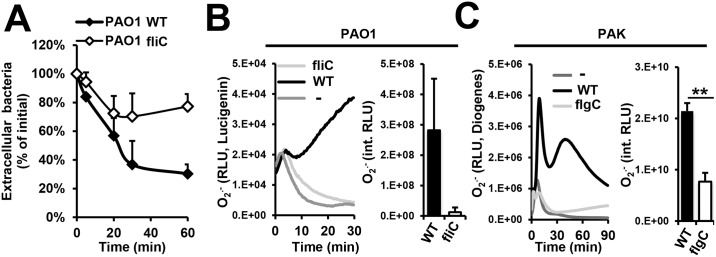
*P*. *aeruginosa* triggers phagocytosis and superoxide production in human PMNs in a flagellum-dependent manner. Human PMNs were exposed to wild-type or flagellum-deficient strains of *P*. *aeruginosa* (10 MOI) for up to 90 min. **(A)** Phagocytosis of PAO1 was followed over time by measuring the decrease in the concentration of living extracellular (non-phagocytosed) bacteria. One representative result, *n* = 3. **(B)** Flagellum-deficient PAO1 *fliC* fails to trigger superoxide production in human PMNs (Lucigenin-enhanced chemiluminescence). Representative kinetics (left) and mean+/-S.E.M (right, *n* = 3) are shown. **(C)** Flagellum-deficient PAK *flgC* also fails to stimulate superoxide release in human PMNs (Diogenes-enhanced chemiluminescence). Representative kinetics (left) and mean+/-S.E.M are shown (right, *n* = 3). **, p<0.01 –Student’s *t*-test.

*P*. *aeruginosa* flagellum is required for phagocytosis by macrophages [50]. Previously, we reported the requirement of a functional cytoskeleton for human PMNs to release NETs triggered by *P*. *aeruginosa* [19]. Based on this, we tested whether the bacterial flagellum is also essential for *P*. *aeruginosa* engulfment by PMNs. Our results in Fig 4A demonstrate that phagocytosis of flagellum-deficient PAO1 is greatly diminished in comparison to its flagellated counterpart.

In addition to phagocytosis, the NADPH oxidase has also been described as a mediator of NET formation induced by different stimuli [26]. Although recently emerging data indicate the existence of NADPH oxidase-dependent and NADPH oxidase-independent mechanisms of NET release, we have previously shown that NET formation stimulated by *P*. *aeruginosa* requires the NADPH oxidase [19, 41]. Therefore, to determine if the flagellum is required for NET formation upstream or downstream of the NADPH oxidase, we measured the neutrophil respiratory burst upon exposure to *P*. *aeruginosa*. Absence of the flagellum results in markedly reduced superoxide production triggered by PAO1 and PAK (Fig 4B and 4C).

Taken together, these results identified that the flagellum plays a key role in *P*. *aeruginosa-*induced NET release via both phagocytosis and NADPH oxidase-mediated superoxide production.

### PMNs do not release NETs in response to *P*. *aeruginosa* flagellin alone

**Fig 5:**
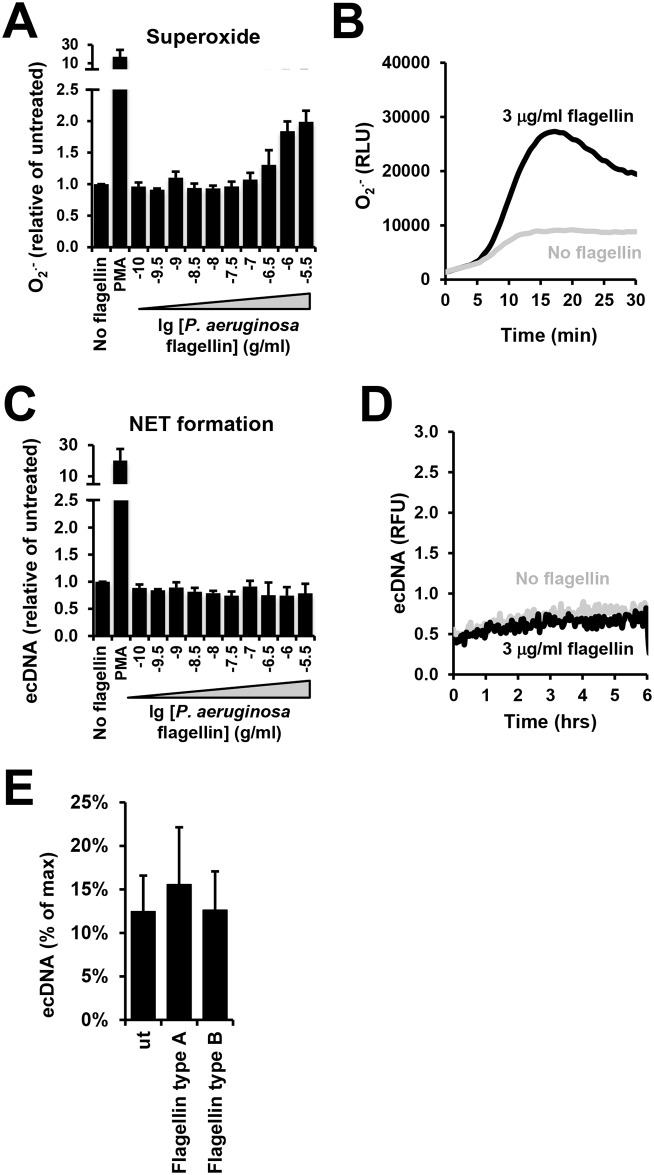
*P*. *aeruginosa* flagellin stimulates superoxide production but does not trigger NET release in human PMNs. Human PMNs were stimulated with a range of commercially available *P*. *aeruginosa* flagellin *in vitro*, and superoxide production [Diogenes-enhanced chemiluminescence: **(A)** summary; **(B)** representative kinetics] and NET formation were measured [Sytox Orange-based release of extracellular DNA: **(C)** summary; **(D)** representative kinetics]. Data are normalized to values without flagellin stimulation. Mean +/-S.E.M., *n* = 3. **(E)** EcDNA release of human PMNs stimulated with 1 μg/ml purified type a and type b *P*. *aeruginosa* flagellin was measured by Sytox Orange fluorescence (mean+/-S.E.M., n = 3). One-way ANOVA and Holm-Sidak’s multiple comparison test. RLU, relative luminescence unit; ns, not significant.

Our data herein established that the flagellum is the main bacterial component of *P*. *aeruginosa* mediating induction of NET release in human PMNs (Figs 2 and 3). To determine the mechanism of this finding, we asked whether purified flagellin, the monomer constituent of flagella, is capable of triggering NET release. As flagellin of other bacterial species including *Listeria monocytogenes* has been shown to stimulate superoxide production in PMNs [54], we first assessed the PMN respiratory burst in the presence of commercially available, recombinant *P*. *aeruginosa* flagellin. Flagellin stimulated PMN superoxide production in the micromolar range in a dose-dependent manner (Fig 5A and 5B). However, same concentrations of flagellin failed to trigger NET release (Fig 5C and 5D) indicating that flagellin alone is not sufficient to induce NET extrusion in human PMNs. We also tested *P*. *aeruginosa* type a and type b flagellins purified from *P*. *aeruginosa* as described [55] and observed no NET release induced by them (Fig 5E). Similarly, purified flagellin of *Shigella flexneri* also failed to induce NET formation in human PMNs (S4 Fig).

### *P*. *aeruginosa*-induced NET release is independent of Toll-like receptor 5 in human PMNs

**Fig 6:**
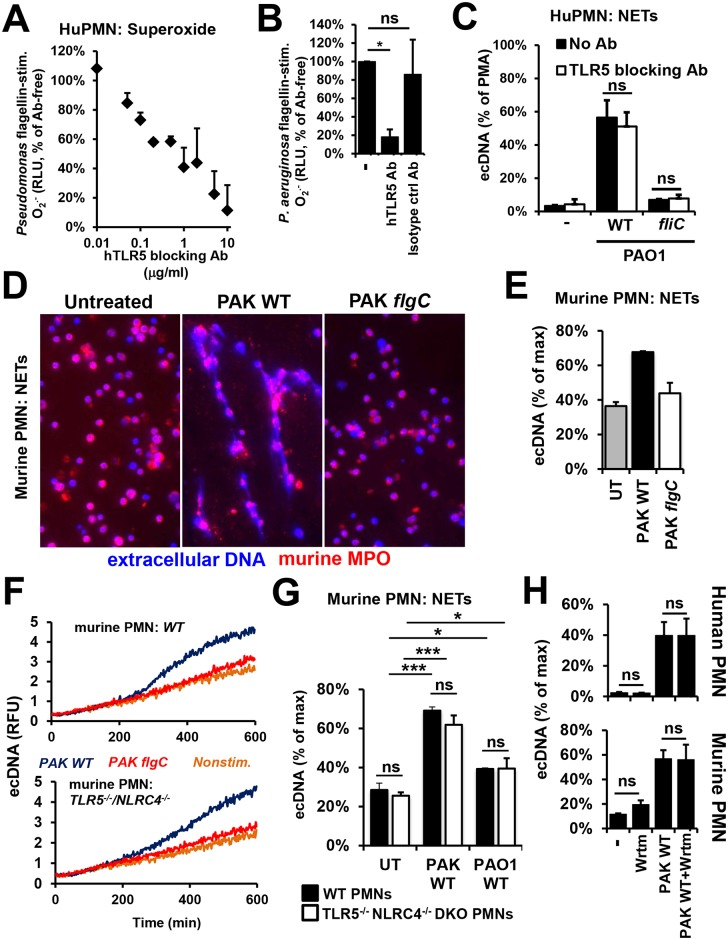
*P*. *aeruginosa*-induced NET formation is independent of TLR5 or NLRC4. **(A)** Human PMNs were stimulated with 1 μg/ml purified *P*. *aeruginosa* PAO1 flagellin in presence of different concentrations of an antibody blocking human TLR5. Superoxide production was measured by Diogenes-enhanced chemiluminescence and was normalized on values without blocking antibody treatment. Mean+/-S.E.M., *n* = 3. **(B)** 10 μg/ml isotype control antibody had no effect on *P*. *aeruginosa* flagellin-stimulated superoxide production. Mean+/-S.E.M., n = 4. **(C)** A concentration of the TLR5-blocking antibody capable of abolishing superoxide production has no effect on *P*. *aeruginosa*-induced NET formation. Extracellular DNA (ecDNA) release in human PMNs stimulated by wild-type (WT) and flagellum-deficient *fliC P*. *aeruginosa* PAO1 (10 MOI) was measured by Sytox Orange fluorescence in the presence or absence of 10 μg/ml TLR5-blocking antibody. Mean+/-S.E.M., *n* = 3. Student’s *t*-test. **(D)** The purity of murine neutrophil preparations was assessed by flow cytometry using Gr-1 as a neutrophil-specific surface marker. One representative experiment, *n* = 13. Cytospin shows a representative image of murine PMNs. **(E)** NET extrusion in murine PMNs triggered by *P*. *aeruginosa* requires the flagellum. Murine bone marrow-derived PMNs were stimulated by wild-type (WT) and flagellum-deficient (*flgC*) *P*. *aeruginosa* PAK (10 MOI), and NET formation was assessed by immunofluorescence: DNA (DAPI, blue), murine MPO (red). One of four independent experiments is shown. **(F)** Wild-type (WT) and TLR5/NLRC4 double KO (DKO) murine PMNs were stimulated with WT and flagellum-deficient (*flgC*) *P*. *aeruginosa* PAK (10 MOI). DNA release was measured by Sytox Orange fluorescence. Representative kinetics are shown, *n* = 4. **(G)** Summary of the previous experiments showing mean+/-S.E.M, *n* = 4. **(H)** The PI3K inhibitor, wortmannin does not affect *P*. *aeruginosa*-induced NET release. Human or murine PMNs were treated with 100 nM wortmannin (Wrtm) for 30 min prior to stimulation with *P*. *aeruginosa* PAK (10 MOI) for 6 hours. EcDNA release was quantitated by Sytox Orange-based fluorescence and is normalized on maximal DNA release value. Mean+/-S.E.M., n = 4. Ns, not significant; WT, wild-type; RFU, relative fluorescence unit; RLU, relative luminescence unit; UT, untreated. *, p<0.05; ***, p<0.001, ANOVA, Tukey-test.

The main surface receptor for extracellular bacterial flagellin is Toll-like receptor 5 (TLR5) that is expressed in PMNs [56, 57]. It is unknown whether TLR5 has any role in NET formation. To assess the potential contribution of the TLR5-flagellin interaction to *P*. *aeruginosa*-induced NET release, we stimulated human PMNs with *P*. *aeruginosa* in the absence or presence of a neutralizing antibody against human TLR5. This antibody inhibits *P*. *aeruginosa* flagellin-stimulated superoxide production in human PMNs in a dose-dependent manner (Fig 6A). The same concentrations of an isotype control antibody had no effect (Fig 6B). Blockade of TLR5 on PMNs with the neutralizing antibody had no effect on NETs expelled in response to *P*. *aeruginosa* PAO1 (Fig 6C). These data suggest that the flagellum mediates *P*. *aeruginosa*-induced NET formation in human PMNs in a TLR5-independent manner.

### Murine neutrophil NET release in response to *P*. *aeruginosa* is flagellum-dependent

Inhibitors and blocking antibodies are currently the only options to experimentally manipulate human PMNs since these cells cannot be genetically modified *in vitro*. Due to the limited experimental repertoire of human PMNs, we also used primary murine PMNs allowing us to test cells obtained from genetically engineered animals. Murine PMNs isolated from bone marrow are capable of releasing NETs both *in vitro [58]* and *in vivo* [59]. We isolated viable, highly pure PMNs from murine bone marrow (S5 Fig). Using fluorescence microscopy, we observed NETs expelled by murine PMNs after exposure to *P*. *aeruginosa* with MPO and DNA co-localization (Fig 6D). Flagellum-deficient *P*. *aeruginosa* did not trigger significant NET release in murine PMNs (Fig 6D–6F). These results confirm similar human PMN data and demonstrate that murine PMNs serve as an excellent model to study the role of flagellum in *P*. *aeruginosa*-induced NET extrusion.

### NET formation in murine PMNs is TLR5- and NLRC4-independent

In addition to TLR5 sensing extracellular flagellum, flagellin in the cytosol is sensed by NOD-like receptor CARD domain containing 4 (NLRC4) [60] that is expressed in PMNs [61]. To further assess whether TLR5/NLRC4-mediated flagellin recognition has any role in NET formation, we subjected murine PMNs expressing (wild-type, WT) or deficient in both TLR5 and NLRC4 (TLR5^-/-^ NLRC4^-/-^ DKO) to flagellated *P*. *aeruginosa*. Flagellated PAK and PAO1 induced NET release in murine PMNs (Fig 6F and 6G). Interestingly, lack of ability to sense flagellin by murine PMNs (TLR5^-/-^ NLRC4^-/-^ DKO) did not affect *P*. *aeruginosa*-induced NET formation (Fig 6F and 6G). This confirms our previous data with human PMNs showing that flagellin recognition pathways are dispensable for neutrophilic deployment of NETs against *P*. *aeruginosa*.

### PI3K is not required for PMNs to release NETs in response to *P*. *aeruginosa*

*P*. *aeruginosa* flagellar motility has been shown to activate the PI3K/Akt pathway to induce phagocytic engulfment [62]. To study whether this pathway is required for NET formation mediated by *P*. *aeruginosa* swimming motility, we used wortmannin to inhibit the PI3K pathway. Inhibiting PI3K had no significant effect on *P*. *aeruginosa*-induced NET formation in neither human, nor murine PMNs (Fig 6H). In our previous study, the same dose of wortmannin significantly inhibited NET formation stimulated by pseudogout-causing calcium pyrophosphate microcrystals suggesting that PI3K involvement in NET release is stimulus-dependent [29].

### Loss of flagellar motility leads to lack of NET induction by *P*. *aeruginosa*

**Fig 7:**
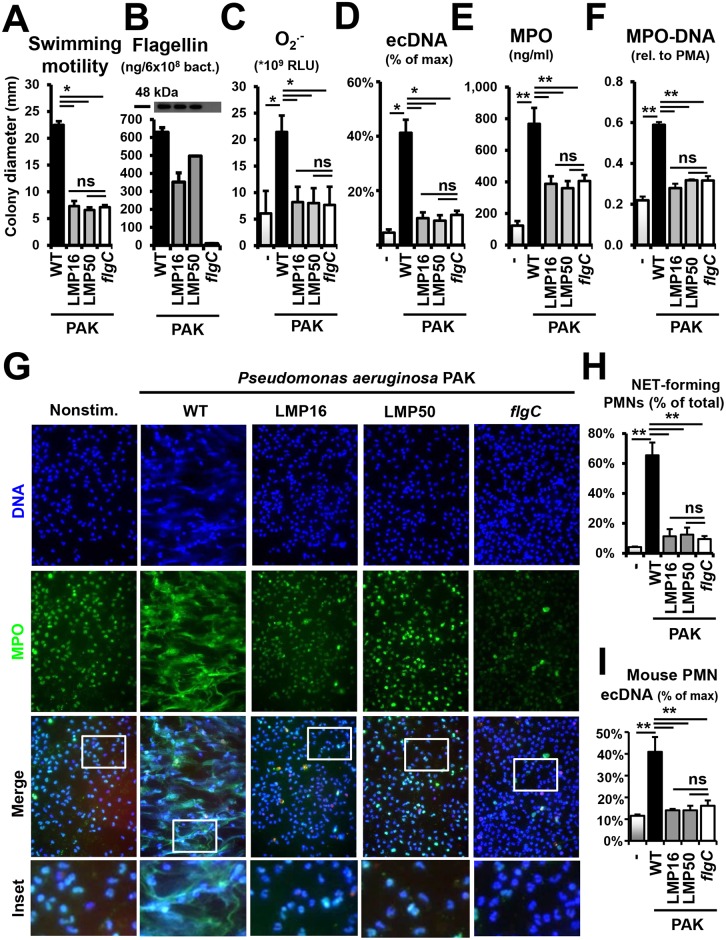
Flagellar motility is essential for *P*. *aeruginosa* to trigger NET release in human PMNs. Human PMNs were stimulated with wild-type (WT, motile, flagellum-expressing), flagellum-deficient (*flgC*, immotile, flagellum-deficient) and flagellar motor-deficient (LMP16 and LMP50, immotile, flagellum-expressing) *P*. *aeruginosa* PAK strains (10 MOI). **(A)** Characterization of swimming motility of PAK strains on semisolid agar. Mean+/-S.E.M, *n* = 3. **(B)** Flagellin expression in PAK mutant lysates measured by western blotting and *P*. *aeruginosa* flagellin ELISA. Blot: One representative result, *n* = 3. ELISA: Mean+/-S.E.M., *n* = 3. **(C)** Neutrophil superoxide production stimulated by PAK strains measured by Diogenes-enhanced chemiluminescence. Mean+/-S.E.M., *n* = 4. **(D)** DNA release in PMNs stimulated by PAK strains assessed as Sytox Orange fluorescence. Mean+/-S.E.M., *n* = 5. **(E)** Release of MPO from human PMNs upon PAK challenge measured by ELISA. Mean+/-S.E.M., *n* = 10. **(F)** NET formation quantitated as release of MPO-DNA complexes induced by PAK strains. Mean+/-S.E.M., *n* = 3. **(G)** Immunofluorescence of NET-releasing human PMNs following PAK challenge: MPO (green), DNA (DAPI, blue). One representative result, *n* = 3. Insets surrounded by white frames show enlarged details of NET formation. **(H)** Immunofluorescence images shown in panel (G) were quantitated by counting NET-forming PMNs and expressing their number as percentage of total. Mean+/-S.E.M., n = 3. **(I)** DNA release in murine PMNs exposed to PAK strains was quantitated by Sytox Orange fluorescence. Mean+/-S.E.M., n = 4. RLU, relative luminescence unit; ns, not significant; PMA, phorbol myristate acetate; MPO, myeloperoxidase. *, p<0.05; **, p<0.01. ANOVA, Tukey test.

The finding of *P*. *aeruginosa*-initiated NET formation being flagellum-dependent but TRL5- and NLRC4-independent (Figs 2–6) could be explained by the fact that the bacterial flagellum not only binds to its host receptors but also confers the ability to swim. Flagellar motility is characteristic during the early exponential phase and is lost at later stages of bacterial growth. Motility is an underappreciated feature of bacterial interactions with the host immune system that is recently gaining recognition [50, 62]. Flagellar motility, not simply flagellum production, has been shown to be a key player in initiating immune responses in macrophages [63, 64]. No study, however, has investigated the role of flagellar motility in PMN activation and NET release. To separate these two functions of the flagellum from each other we took advantage of *P*. *aeruginosa* mutants deficient in genes responsible for propulsion of the flagellum [51]. The *P*. *aeruginosa* flagellum is powered by a complex bacterial motor consisting of multiple proteins encoded by two sets of homologous motor genes: 1) *motA*, *motB* and 2) *motC*, *motD* [51, 65]. Disruption of both loci is required to completely abolish swimming motility; deletion of either set of operons is not sufficient to eliminate swimming [51, 65]. We characterized motility and flagellin production in 2 mutant PAK strains deficient in the following *mot* genes: strain LMP16 (Δ*motCD motB)* and strain LMP50 (Δ*motAB motD)* [51]. As expected, swimming motilities of the flagellar motor-deficient strains (LMP16 and LMP50) and the flagellum-deficient (*flgC*) mutant were abolished (Fig 7A). On the other hand, flagellin production (measured by western blotting and ELISA in bacterial lysates) was only missing in the *flgC* strain (Fig 7B). Flagellar motor-deficient mutants produced flagellin to an extent similar to that of the wild-type (WT) strain (Fig 7B). Together, these data demonstrate that the flagellar motor-deficient strains produce a flagellum that is paralyzed for rotation; they have impairment in swimming motility but express normal levels of flagellin.

To assess the potential role of flagellar motility in NET release induced by bacteria, we stimulated human PMNs with wild-type, flagellum-deficient and flagellar motor-deficient *P*. *aeruginosa* PAK strains to measure NADPH oxidase activity and NET release. Superoxide production was abolished in the absence of both flagellar motility and flagellum production (Fig 7C): nonstimulated (6.08+/-4.24), PMA (57.25+/-7.08), PAK WT (21.44+/-3.10), LMP16 (8.21+/-2.89), LMP50 (8.03+/-2.81) and *flgC* (7.68+/-3.43) (*10^9^ RLU, mean+/-S.E.M., *n* = 4). Similarly, *P*. *aeruginosa*-induced ecDNA release was significantly reduced when motility was abolished (Fig 7D): 81.8+/-3.6% reduction with LMP16, 85.8+/-4.6% with LMP50 and 88.6+/-4.1% with *flgC* (mean+/-S.E.M., *n* = 5). PAK-induced MPO release showed a similar pattern (Fig 7E). Wild-type PAK triggered 767.6+/-101.0 ng/ml MPO release, whereas flagellum-deficient *P*. *aeruginosa* induced considerably less MPO release of 405.8+/-37.7 ng/ml (mean+/-S.E.M., *n* = 10) (Fig 7E). Immotile, flagellum-expressing PAK mutants induced PMN behavior similar to that of the flagellum-deficient strain: 387.9+/-47.9 ng/ml MPO release by LMP16 and 360.0+/-47.9 ng/ml by LMP50 strains (Fig 7E, mean+/-S.E.M., *n* = 10). Of note, a significant portion of *P*. *aeruginosa*-stimulated MPO release, unlike other measures, is independent of the flagellum (Fig 7E). These data confirm our previous observations that NET formation is the main, but not the only, mechanism to mediate MPO release from human PMNs stimulated by *P*. *aeruginosa* [19].

The fact that a motile flagellum provides the main mechanism of *P*. *aeruginosa*-triggered NET extrusion was further confirmed by our MPO-DNA ELISA data, showing greater amounts of MPO-DNA complexes released in response to motile *P*. *aeruginosa* (Fig 7F). NET inductions by *P*. *aeruginosa* were reduced: by 84.5+/- 7.4% (LMP16), by 73.3+/-3.5% (LMP50) and by 73.2+/-3.7% (*flgC*) (mean+/-S.E.M., *n* = 3) (Fig 7F). Immunofluorescence staining of NETs following stimulation with wild-type and PAK mutants confirmed these data qualitatively (Fig 7G and 7H). As reported previously, MPO co-localized with extracellular DNA in PAK-induced NETs (Fig 7G). Human PMNs exposed to flagellum-deficient or flagellar motor-deficient *P*. *aeruginosa* strains released only minimal amount of NETs (Fig 7G). Murine PMNs exposed to the same PAK mutants exhibited an identical pattern of NET release (Fig 7I).

Taken together, the results presented in Fig 7 indicate that flagellar motility, and not flagellum production alone, is the main factor of NET release, in the presence of flagellated *P*. *aeruginosa*.

### Forced contact between immotile bacteria and PMNs restores their ability to induce NET release

**Fig 8:**
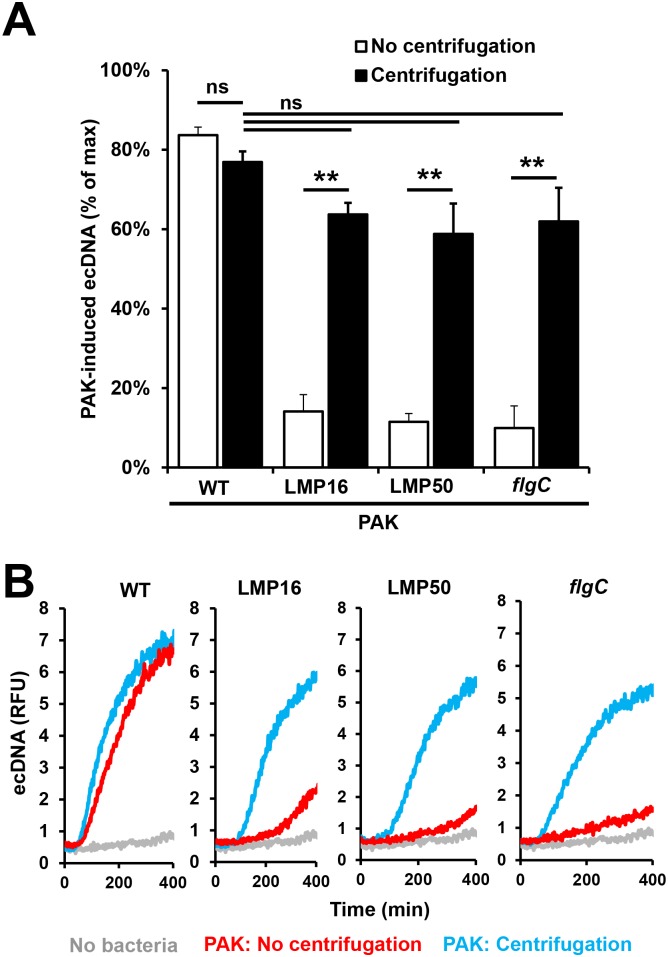
Forced contact of immotile *P*. *aeruginosa* strains with human PMNs restores their ability to trigger NET release. Human PMNs were exposed to wild-type (WT, motile, flagellum-expressing), flagellum-deficient (*flgC*, immotile, flagellum-deficient) or flagellar motor-deficient (LMP16 and LMP50, immotile, flagellum-expressing) *P*. *aeruginosa* PAK strains (10 MOI) with or without centrifugation (2,000 g 5 min). DNA release was quantitated in the presence of Sytox Orange for 6 hours. **(A)** Summary or **(B)** representative kinetics of three independent experiments are shown (mean+/-S.E.M.). One-way ANOVA and Holm-Sidak's multiple comparisons test. **, p<0.01; ns, not significant.

To support our previous findings we centrifuged wild-type, flagellum- and flagellar motor-deficient strains of *P*. *aeruginosa* on human PMNs and measured NET release. Centrifugation of bacteria on PMNs bypasses the need for motility to establish cell-cell contact. All four immotile bacterial strains were capable of inducing close to maximal DNA release in human PMNs upon centrifugation on PMNs (Fig 8A and 8B). NET release by the wild-type bacterium was not affected by centrifugation (Fig 8A and 8B). To confirm that live bacteria are required to induce NET formation, human PMNs were stimulated with heat-killed *P*. *aeruginosa* and superoxide production and DNA release were measured. Both readouts were inhibited by the heat-treatment indicating that live bacteria are needed to induce maximal NET formation (S6 Fig). Thus, bypassing the requirement for motility to enable live *P*. *aeruginosa*-PMN contact restores the ability of immotile bacterial strains to trigger NETs.

### Both motility gene loci are essential for *P*. *aeruginosa* to induce maximal NET release

**Fig 9:**
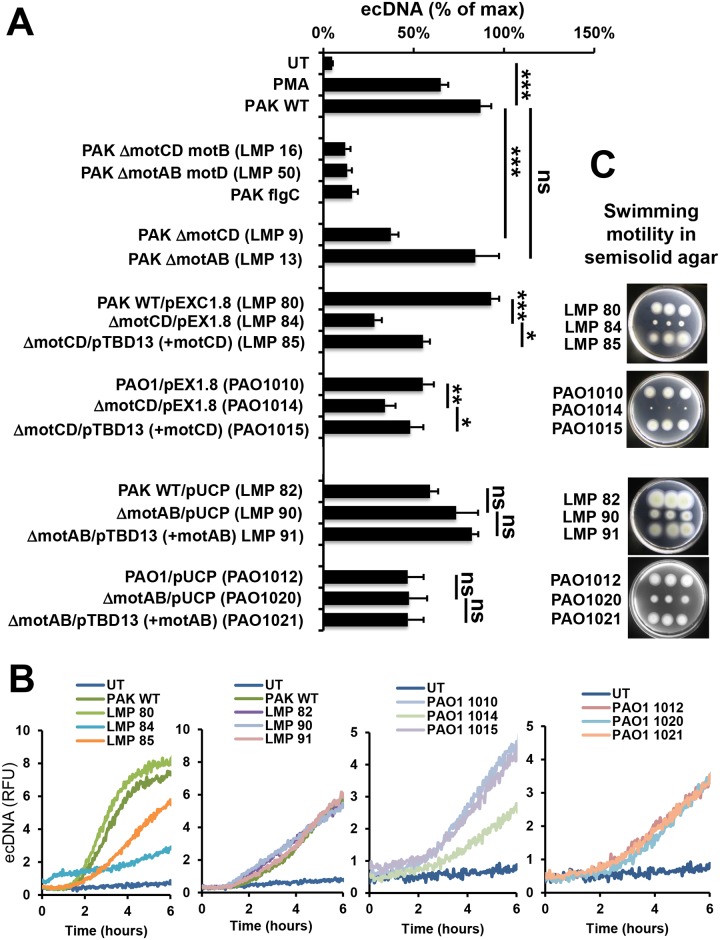
Disruptions of genes in both *mot* gene loci are required to abolish *P*. *aeruginosa*-induced NET release. *P*. *aeruginosa* strains deficient in one of the two motility gene operons and their complemented derivatives were tested. Human PMNs were stimulated with indicated strains of *P*. *aeruginosa* PAK or PAO1 and DNA release was measured using Sytox Orange for 6 hrs (shown as % of max). **(A)** Averages or **(B)** representative kinetics of four independent experiments performed on PMNs isolated from independent donors are shown. The nonmotile PAK strains having mutations in both motor loci are shown for comparison (LMP16, LMP50). *MotCD*-deficient *P*. *aeruginosa* (LMP84, PAO1014) strains trigger significantly less NET release than their wild-type counterparts (LMP80, PAO1010). Complementation of the *motCD* genes results in significant increase in NET induction in both backgrounds (LMP85, PAO1015). *MotAB*-deficient strains (LMP90, PAO1020) and their complemented derivatives (LMP91, PAO1021) trigger NET release that is not different from that stimulated by wild-type bacteria (LMP82, PAO1012). **(C)** Swimming motility in soft agar plates is shown for the complementation strains. WT, wild-type; RFU, relative fluorescence unit; UT, untreated, no stimuli. One-way ANOVA and Holm-Sidak’s multiple comparisons test. *, p<0.05; **, p<0.01; ***, p<0.001; ns, not significant.

To assess which motility genes are required for *P*. *aeruginosa* to induce NET formation in human PMNs, we tested PAK strains deficient in either one of the *motAB* or *motCD* loci [51]. As shown in Fig 9, *motCD*-deficient *P*. *aeruginosa* (LMP9, LMP84 and PAO1014) exhibited significant impairment in inducing NET formation while *motAB*-deficient bacteria (LMP13, LMP09 and PAO1020) remained largely unaffected. These data suggest that the *motCD* genes are more crucial for inducing NETs. However, NET release triggered by Δ*motCD* PAK was still higher than that induced by the completely immotile bacteria that have lesions in both loci, suggesting that a fully functional motor complex is needed to trigger maximal NET formation (Fig 9).

### Complemented motor-deficient *P*. *aeruginosa* enhances NET formation

Next we aimed at restoring the impaired NET-inducing ability of *motAB*-deficient bacteria by cloning and reintroducing functional *motAB* genes in both PAK and PAO1 backgrounds. The following PAK strains were created: wild-type PAK transformed with empty vector (LMP 80), *motCD*-deficient PAK with the vector (LMP84) and *motCD*-deficient PAK complemented with functional *motCD* (LMP85) (Table 1).

**Table 1:** Bacterial strains and plasmids used in this study.

Strain name	Description	Reference
***PAK strains***		
**PAK**	Wild type	Pathogenesis Corp.
**LMP9**	ΔmotCD::Gen^r^	[51]
**LMP13**	ΔmotAB::Gen^r^	[51]
**LMP16**	motB::Tn5 (Tet^r^) ΔmotCD::Gen^r^	[51]
**LMP50**	ΔmotAB::Tet^r^ motD::Gen^r^	[51]
**LMP57**	flgC1::Tn5(Tet^r^)	Pathogenesis Corp. (AKA 056E02)
**LMP80**	PAK/pEX1.8	This work
**LMP82**	PAK/pUCP-NcoI	This work
**LMP84**	LMP9/pEX1.8	This work
**LMP85**	LMP9/pTBD13 (motCD^+^)	This work
**LMP90**	LMP13/pUCP	This work
**LMP91**	LMP13/PTBD33 (motAB^+^)	This work
***PAO1 strains***		
**MPAO1**	Wild type PAO1	From C. Manoil; [66]
**fliC**	fliC::ISlacZ	PA1092-G03::lacZbp01q1; [66]
**MPAO1001**	ΔmotCD	derived from MPAO1; [51]
**MPAO1002**	ΔmotAB	derived from MPAO1; [51]
**MPAO1010**	MPAO1/ pEX1.8	This work
**MPAO1012**	MPAO1/ pUCP-NcoI	This work
**MPAO1014**	MPAO1001/ pEX1.8	This work
**MPAO1015**	MPAO1001/ pTBD13 (motCD^+^)	This work
**MPAO1020**	MPAO1002/ pUCP-NcoI	This work
**MPAO1021**	MPAO1002/ pTBD33(motAB^+^)	This work
***Plasmids***		
**pEX1.8**	Complementation vector	GenBank:JQ342676.1 ApR (Carbenicillin 300 μg/ml); [67]
**pUCP-NcoI**	Complementation vector	ApR (Carbenicillin 300 μg/ml); [68]
**pTBD13**	motCD^+^ in pEX1.8	1.8 kb insert; primers: GCCTCCGATGGCCACCCACGTGACGCG
		CTCTTCCTGGCATGGGTCTACCCGGTTGG
**pTBD33**	motAB^+^ in pUCP-NcoI	Insert 5.9kb with native promoter; primers:
		ATCGTCCAGCGCCAGGTGGGTGTTGCCCTTCT ATCCGCGCGGATGCGCAGGGCGCGAG

Gene nomenclature—adopted from reference [69]: *motAB* designates PA4954/4953 and *motCD* refers to PA1460/1461.

Similar PAO1 strains were generated: PAO1 wild-type containing empty vector (PAO1010), *motCD*-deficient PAO1 containing vector (PAO1014) and *motCD*-deficient PAO1 complemented with *motCD* genes (PAO1015) (Table 1). *MotCD*-deficiency lead to significantly impaired induction of NET release; whereas introduction of functional *motCD* genes resulted in increased NET release in both PAK and PAO1 backgrounds (Fig 9A and 9B). Similar pattern was observed with superoxide production (S7 Fig).

Complemented strains were also created for the *motAB* mutants: PAK wild-type transformed with empty vector (LMP82), *motAB*-deficient PAK (LMP90) and *motAB*-deficient PAK complemented with functional *motAB* genes (LMP91); PAO1 wild-type transformed with empty vector (PAO1012), *motAB*-deficient mutant (PAO1020) and *motAB*-deficient strain complemented with functional *motAB* genes (PAO1021) (Table 1). Consistent with previous data, *motAB* mutants did not result in impairment in NET-induction and there were no significant differences between the *motAB*-deficient strain and its complemented derivatives in each background (Fig 9A and 9B). Swimming motility of the strains was characterized in semisolid motility agar (Fig 9C). Both, *motAB*- and *motCD*-deficiencies led to significant losses of swimming motility in semisolid agar while complemented derivatives regained swimming motility (Fig 9C).

Thus, our experiments suggest that the *motCD* genes play a primary role in determining the extent of NET induction by planktonic *P*. *aeruginosa* but both sets of motor genes are required for maximal NET-induction.

## Discussion

The purpose of this study was to identify the main bacterial component of *P*. *aeruginosa* triggering NET formation and to gain insight into its mechanism. *P*. *aeruginosa* is an opportunistic pathogen representing a serious medical problem. PMNs, the primary immune cells fighting this bacterium, release large amounts of NETs when challenged with *P*. *aeruginosa* [19, 33, 41, 45]. These NETs then kill and trap bacteria [17]. NETs, however, also cause tissue damage in airway diseases characterized by *P*. *aeruginosa* infections (e.g., CF and COPD) [31, 32, 70]. Therefore, it is important to illuminate the cellular-molecular details of *P*. *aeruginosa*-induced NET formation to better understand its clinical relevance in various disorders. Our data show that early growth-phase bacteria are the strongest NET-inducers. Our data identify flagellum as the main component of bacteria triggering NETs, thereby filling in a major gap in our understanding of the molecular details of bacterium-triggered NET formation. Until now, only two bacterial components (LPS and pyocyanin) were described as weak NET-inducers [17, 40]. Our detailed characterization documents the major contribution of the flagellum to *P*. *aeruginosa*-induced NET extrusion in both human and murine PMNs, thus adding a new mechanism to the proinflammatory repertoire of flagellum [71].

Detection of *P*. *aeruginosa* flagellin was traditionally performed by immunoblotting or electron microscopy [72]. Neither of these methods is, however, suitable for accurate absolute quantitation. The new ELISA assay established here enables easier detection of varying *P*. *aeruginosa* flagellin levels in a large number of samples. The highly reproducible standard ensures accurate absolute quantitation of flagellin levels that is vital to study *P*. *aeruginosa* flagellum interactions within host cells including PMNs.

TLR5 is expressed on airway epithelial cells and several innate immune cell types including PMNs [56, 73]. TLR5 is the main receptor mediating activation of airway epithelial cells of *P*. *aeruginosa* via flagellin recognition [73] and the TLR5-flagellin interaction is a major mediator of airway inflammation in CF [74]. TLR5 also acts as a modifier gene in CF [74]. Therefore, it was very surprising to observe that *P*. *aeruginosa*-induced NET release is independent of TLR5 in both human and murine PMNs. In further support of this finding, we also show that recombinant *P*. *aeruginosa* flagellin monomers are capable of stimulating NADPH oxidase activity without inducing NET release. Flagellin monomers bind to and activate TLR5 [57]. Thus, our data suggest that the flagellum mediates *P*. *aeruginosa*-induced NET release by a novel mechanism independent of the flagellum-sensing machinery of PMNs (TLR5/NLRC4). Moreover, we found that flagellum-mediated swimming motility is the key mediator of NET release. These data add to currently published reports emphasizing the importance of swimming motility in *P*. *aeruginosa* virulence in macrophages [50, 62] and imply that flagellum also contributes to bacterial virulence by mechanisms other than activation of the TLR5 signaling pathway. Motility-mediated bacterial virulence mechanisms represent a large gap in the scientific literature and require more detailed studies. It cannot be automatically assumed that motile bacterial pathogenesis is entirely the result of direct flagellum-receptor interactions. Instead, we must consider that other motility-based mechanisms can also take place. The presence of a rotating flagellum enables *P*. *aeruginosa* to swim which significantly increases the chances for bacterium-neutrophil encounters. It is very likely that pattern recognition receptors other than TLR5 and NLRC4 are responsible for direct binding of *P*. *aeruginosa* to PMNs during initiation of NET extrusion. The identities of these receptors remain to be elucidated.

The polar flagellum of *P*. *aeruginosa* is powered by a complex motor containing dual stator units, MotAB and MotCD [reviewed in [75]]. The stators generate the torque used to turn the flagellar rotor. In liquid medium, either stator is sufficient to power the flagellum as deletion of either the *motAB* or *motCD* locus has little effect on swimming speed and only the deletion of both loci renders the bacterium immotile [51, 75]. However, under conditions requiring higher torque, for example in semi-solid motility agar as (shown in Fig 9C) or on a swarming motility plate, the MotCD stator plays the dominant role [51, 69]. Results presented here reveal that the *motCD* mutants strongly impair *P*. *aeruginosa*-induced NET formation, *motAB* mutants have little effect, and mutants with defects in both *motAB* and *motCD*, are most severely impaired for NET release induced in human PMNs. These findings suggest that motility is key to trigger NET formation. Moreover, they suggest that the stator that can provide the highest torque to the rotor is more important in NET formation. In line with previous studies, our results also suggest that flagellar motility genes could provide novel targets of pharmaceutical intervention to intervene with *P*. *aeruginosa* motility as a virulence mechanism currently gaining recognition.

A flagellum is typically expressed in environmental isolates of *P*. *aeruginosa* and early clinical isolates of CF patients [76, 77]. Loss of the flagellum is one of the characteristic changes accompanying the adaptation of *P*. *aeruginosa* in CF airways [76–80]. In chronic CF patients, *P*. *aeruginosa* mainly exists in biofilms [81]. However, biofilms are dynamic structures, and motile, flagellated bacteria likely break free from biofilms, possibly interacting with PMNs and shedding flagella. This is supported by recent data showing that *P*. *aeruginosa* flagellin is detected in sputa of chronic CF patients [82]. Thus, we speculate that the mechanism described here not only can have clinical relevance in early but also in chronic CF airway disease.

Our studies provide a potential, novel explanation as to why it is advantageous for *P*. *aeruginosa* to lose its flagellar motility early on in colonization of the airways in CF. PMNs and NETs could provide a significant external pressure for *P*. *aeruginosa*’s down-regulation of flagellin expression in CF airways. This is supported by published data showing that NE cleaves flagellin and down-regulates flagellum expression in *P*. *aeruginosa* [83, 84]. Loss of flagella and swimming motility could be the primary mechanism by which late-phase and mucoid CF isolates of *P*. *aeruginosa* acquire resistance against NET-mediated killing [33, 41, 45, 85].

It is important to note that while *P*. *aeruginosa*-induced NET release is almost entirely flagellum- and flagellar motility-dependent, total MPO and HNE release is only partially dependent on these factors. This is in line with our previous observations stating that NET formation is the main, but not the only, mechanism of MPO and HNE release from PMNs challenged with *P*. *aeruginosa* [19, 41]. Excessive degranulation could potentially be responsible for the NET-independent release of these primary granule components [86, 87].

Our results demonstrating that murine PMNs also expel NETs in response to *P*. *aeruginosa* in a flagellum-dependent but TLR5-independent manner confirm the usefulness of murine PMNs as a model to study the mechanism of NET release stimulated by planktonic forms of bacteria. Having established murine NET measurements enables us to test *ex vivo* NET release in genetically modified murine PMNs. This genetic approach complements results obtained with human PMNs that are not suitable for genetic modifications.

Our data show that early growth-phase planktonic bacteria are the strongest NET-inducers in human PMNs. Although the idea that planktonic bacteria can induce NET release has previously been challenged [88], our observation is in line with numerous articles published by several independent groups all reporting robust NET release induced by (planktonic or individual) bacteria of a wide variety of different species [19, 33, 41, 45, 89–91]. That said, it is very likely that different mechanisms are responsible for NET release induced by bacteria or large microbes (fungal hyphae) [88]. Although it does not form the focus of the current study, but our data showing that flagellum is required for both phagocytosis and NET release and bacterium-PMN contact is essential to induce NET formation, indicate that phagocytosis of *P*. *aeruginosa* is necessary for NET release. This idea suggests that the same PMN can engulf bacteria and undergo subsequent NET release, as well. Previously, it has been proposed that a single PMN either performs phagocytosis or undergoes NET formation but not two functions in one cell [88]. Likely, the primary response of PMNs to planktonic bacteria is phagocytosis that is followed by NET release once uptake of more microbes is not feasible. This mechanism has already been proposed earlier [26] and is also supported by our data. Future focused studies need to be performed to understand the very exciting question what factors determine PMN effector mechanisms and cell fate in response to bacteria. It is highly important to address this problem to learn about the unanswered questions of NET formation [92], to understand what leads to unnecessary PMN activation in several diseases [93] and to be able to develop novel PMN-based therapies [94].

Overall, the results presented here reveal a novel proinflammatory mechanism of the bacterial flagellum and identify it as the main factor of flagellated bacteria triggering NET formation. We also identified flagellar motility as its primary mechanism to mediate *P*. *aeruginosa*-induced activation of PMNs that likely occurs in CF airways, contributes to disease pathogenesis and possibly points to a new, future therapeutic target.

## Materials and Methods

### Ethics statement

The Institutional Review Board of the University of Georgia approved the human subject study to collect peripheral blood from volunteers anonymously (UGA# 2012-10769-06). Enrolled healthy volunteers were non-pregnant and heavier than 110 pounds without any infectious disease complication. All adult subjects provided informed consent, and no child participants were enrolled into the study. The studies were performed following the guidelines of the World Medical Association's Declaration of Helsinki.

The Institutional Animal Care and Use Committees (IACUC) of the University of Georgia and the Georgia State University reviewed and approved the mouse protocols used in this study: UGA IACUC protocols: A2012 11-003-Y3-A3, A2014 08-019-Y2-A0 and GSU IACUC protocol: A14033. All animal experiments were performed in accordance with NIH guidelines, the Animal Welfare Act and US federal law. Animals were housed in centralized research facilities accredited by the Association of Assessment and Accreditation of Laboratory Animal Care International.

### Human neutrophil isolation from venous blood

Human PMNs were isolated as described previously [19, 41]. Briefly, whole blood was drawn at the Health Center of the University of Georgia from volunteers. Coagulation was prevented with heparin. Red blood cells were removed by Dextran sedimentation (GE Healthcare), and PMNs were separated using Percoll gradient centrifugation. Cell viability was determined by Trypan blue dye extrusion (>98%). Neutrophil purity was assessed by cytospin preparations and flow cytometry. Autologous serum was prepared from coagulated blood by centrifugation and sterile filtration. Calcium- and magnesium-containing HBSS (Mediatech, Manassas, VA, USA) supplemented with 1% autologous serum, 5 mmol/l glucose and 10 mmol/l HEPES was used as the assay buffer.

### Mice

Wild-type (WT) C57BL/6 mice were purchased from Jackson Laboratories and maintained in the animal facility of the College of Veterinary Medicine, University of Georgia, Athens. 10-15-week-old mice were used throughout the study. TLR5/NLRC4 double gene-deficient mice on a C57BL/6 background were kept in the Georgia State University animal facility. TLR5KO mice used here were originally generated by Dr. Shizuo Akira (Osaka University, Osaka, Japan) [95] and backcrossed/maintained as previously described [96]. NLRC4 KO mice generated on a pure C57BL/6j background were kindly provided by Genentech (Genentech, Inc. South San Francisco, CA) [97]. Age- and sex-matched healthy C57BL/6 mice were used as controls. Mice were euthanized on the day of the experiment by CO_2_ asphyxiation and cervical dislocation according to University of Georgia and Georgia State University IACUC guidelines.

### Murine neutrophil isolation

Murine bone marrow-derived PMNs were collected from femur and tibia. Bones were flushed with RPMI-1640 medium (Corning, Manassas, VA) and washed in sterile PBS. 1 ml AKC buffer (Lonza, Walkersville, MD) was used to lyse red blood cells. Cells were passed through a 40 μm pore size Nylon Mesh strainer (Fisherbrand, Fisher Scientific, Pittsburgh, PA, USA) and subsequently washed twice with and re-suspended in sterile PBS before being layered on top of a two-step Percoll (Sigma-Aldrich, St. Louis, MO, USA) gradient (62% and 81%), as described previously [98]. After centrifugation (1600 g, 30 min), PMNs accumulated at the interface of 81% and 62% Percoll layers were collected and washed twice in sterile PBS. Cell numbers were determined with a hemocytometer. Cell viability determined by Trypan Blue exclusion was always higher than 98%. Neutrophil purity was also periodically confirmed with hematoxylin and eosin (H/E) staining (Sigma, St. Louis, MO, USA) of cytospins prepared using CytopsinTM Cytocentrifuge (ThermoScientific, Waltham, MA, USA) (S5C Fig).

### Flow cytometry

The purity of the murine neutrophil preparations was routinely confirmed via flow cytometry (LSRII, BD Technologies). Anti-Gr-1 antibody (Miltenyi Biotec, San Diego, CA, USA) was used against Gr-1-expressing granulocytes following the manufacturer’s recommendations. Cells were analyzed in BD LSRII flow cytometer (BD Biosciences, San Jose, CA, USA) using BD FACSDiva 6.0 software (BD Biosciences, San Jose, CA USA) at the Imaging Core Facility of the Department of Infectious Diseases at UGA. The described protocol resulted in more than 95% PMNs (S5 Fig).

### *Pseudomonas aeruginosa* strains

The following *Pseudomonas aeruginosa* strains were used in this study. The PAO1 parental strain (wild-type, WT) was MPAO1 and its flagellum-deficient PAO1 mutant (strain ID: 245, genotype: PA1092-G03::lacZbp01q1, referred to as “*fliC*”) were obtained from the *Pseudomonas aeruginosa* PAO1 transposon mutant two-allele library (University of Washington, Seattle, WA; Manoil laboratory) established using NIH funds (grant#: P30 DK089507) [66]. The PAK wild-type (WT) and flagellum-deficient mutant *flgC1*::Tn*5* were obtained from Pathogenesis Corporation (referred to as “*flgC*”). The flagellar motor mutant strains were described elsewhere (51). Strains are listed in Table 1 that also includes the complementation plasmids [67, 68]. The gene designations were revised to be consistent within the *Pseudomonas aeruginosa* field and refer to PA1460-61 (*motCD*) and PA4954-53 (*motAB*) [51]. *P*. *aeruginosa* strains were cultured in Luria-Bertani broth for the indicated periods of time. Bacteria were washed twice in PBS and resuspended in calcium- and magnesium-containing HBSS. Bacterial cultures were set to an optical density (OD) = 0.6 at 600 nm in 96-well microplates measured using a Varioskan Flash combined microplate reader (ThermoScientific, Waltham, MA USA). This corresponds to a bacterial density of 10^9^/ml, as determined by serial dilutions and colony forming unit (CFU) assays [40, 99]. In some experiments, optical density of bacterial cultures was followed over time in an Eon Microplate Spectrophotometer (BioTek, Winooski, VT) to record kinetic growth curves (Fig 1A).

### PCR for PAO1 genotyping

The genotype of the PAO1 *fliC* mutant was confirmed by PCR using the primers and conditions suggested on the web site of the PAO1 two-allele library. PAO1 WT and *fliC* bacteria were cultured overnight, washed twice in HBSS and set to an OD = 0.6 as described above. 100,000 bacteria in 2 μL distilled water were added to the PCR reaction mix and served as a DNA template. The PCR mix (20 μl reaction volume) contained 10 mM dNTP (Life Technologies, Carlsbad, CA), 50 mM MgCl_2_, 10 μM forward and reverse primers and Taq DNA Polymerase (Life Technologies, Carlsbad, CA). The following gene-specific primers were used: *fliC* (F: 5’- TGCAGCAGTCCACCAATATC-3’; R: 5’- GTTGGTAGCGTTTTCCGAGA -3’, product size: 1081 bp), *pilA* (F: 5’- GGAATCAACGAGGGCACC -3’; R: 5’- ACCCAGTTTCCTTGATCGTG -3’, product size: 865 bp). PCR reaction parameters were: 94°C for 0.5 min, followed by 35 cycles of 94°C for 30 sec, 60°C for 1 min and 68°C for 90 sec. The PCR reaction was carried out in a Biometra PCR thermocycler (Biometra, Göttingen, Germany). *PilA* was used as loading control. The PCR products were resolved on 2% agarose gel and stained with Gelstar DNA stain (Lonza, Walkersville, MD, USA). The genotype of the flagellum-deficient PAO1 *fliC* strain was confirmed by PCR (S1C Fig). Lack of contaminating DNA was confirmed by PCR without template (no bacteria) (S1C Fig).

### Bacterial motility assay

For the swimming motility assay, bacteria were grown overnight, washed twice in HBSS and set to an optical density (OD) = 0.6 as described previously. 10 μL of bacterial cultures were spotted on the center of freshly prepared LB+0.3% agar plates and incubated at room temperature. After 48 hours, diameters of colonies were measured and expressed in millimeters (mm) [100]. For complementation experiments, the strains were streaked for single colonies on LB medium with 300ug/ml carbenicillin. Three single colonies were toothpicked into tryptone motility agar containing 10 g/L tryptone 5 g/L NaCl and 3 g/L agar. Plates were incubated overnight and photographed.

### *P*. *aeruginosa* flagellin

Flagellin of *P*. *aeruginosa* was obtained from two independent sources. First, purified *P*. *aeruginosa* flagellin was purchased from Invivogen (San Diego,CA, USA). Flagellin obtained from this commercial source is extracted by acid hydrolysis and is purified by ultrafiltration and chromatography. The identity of the *P*. *aeruginosa* strain and the type of the flagellin were not revealed by the company. Second, *P*. *aeruginosa* flagellin was also obtained as a kind gift from Dr. Gerald Pier (Massachusetts General Hospital, Boston, MA). Recombinant *P*. *aeruginosa* flagellins were purified from *E*. *coli* expressing His-tagged type a or b *fliC* genes as described previously [55, 101].

### Quantitation of extracellular DNA release

DNA release from human PMNs was quantitated as described [19, 29]. Briefly, 250,000 PMNs were seeded on 96-well black transparent bottom plates in the presence of 0.2% Sytox Orange (Life Technologies, Grand Island, NY, USA) membrane-impermeable DNA-binding dye. PMNs were infected with 10:1 multiplicity of infection (MOI) *P*. *aeruginosa* as indicated. Fluorescence (excitation: 530 nm, emission: 590 nm) was recorded for up to 8 hrs in a fluorescence microplate reader (Varioskan Flash, ThermoScientific, Waltham, MA, USA) at 37°C. DNA release is expressed as % of the maximum obtained by saponin-mediated (1 mg/ml; Sigma-Aldrich, St. Louis, MO, USA) neutrophil lysis and DNA exposure.

### Immunofluorescence staining of myeloperoxidase and citrullinated histone H4

Immunofluorescence staining of human MPO and citrullinated H4 was performed as previously described [19, 29, 40, 41]. Briefly, adherent murine or human PMNs were exposed to different strains of *P*. *aeruginosa* (10 MOI, 3 hrs, 37°C). After incubation, fixed and permeabilized samples [4% paraformaldehyde (Affymetrix, Celeveland, OH)] were blocked with 5% Normal Donkey serum (Sigma-Aldrich, St. Louis, MO, USA, in PBS) for 30 min at room temperature. Fixed human NETs were incubated with monoclonal mouse anti-human myeloperoxidase/FITC antibody (1:500, Dako, Clone MPO-7) and polyclonal rabbit anti-histone H4 (citrulline 3) (1:1000, Millipore, Billerica, MA) overnight at 4°C. The citrullinated histone staining requires the use of a secondary antibody after three washes: Alexa Fluor 594-labelled donkey anti-rabbit secondary antibody for 1 hr (1:2000, Molecular Probes, Grand Island, NY). Murine PMNs were first stained with goat anti-mouse MPO antibody (R&D Systems, Minneapolis, MN, 1:1,000) overnight at 4°C, followed by staining with FITC-labelled, donkey anti-goat IgG (Jackson ImmunoResearch, West Grove, PA, USA, 1:800, 1 hr, dark). DNA was stained with DAPI (2 min, room temperature, 1:20,000, Molecular Probes, Grand Island, NY). Specimens were washed three times with PBS containing 0.1% Tween-20 (Sigma-Aldrich, St. Louis, MO, USA) between each step. Mounted specimens were analyzed with Zeiss AxioCam HRM fluorescence microscope Axioplan2 imaging software. NET formation was quantitated by counting at least 200 PMNs per sample and by determining the proportion of NET-forming cells compared to the total population.

### MPO, HNE ELISA

Concentration of human MPO in PMN supernatants was quantitated by commercial ELISA kit (R&D Systems, Minneapolis, MN, USA) as previously described [19, 41]. Human neutrophil elastase release was assessed by sandwich ELISA: diluted supernatants were applied to 96-well high binding microloan ELISA plates (Greiner bio-one, Germany) pre-coated overnight with anti-human neutrophil elastase rabbit polyclonal antibody (1:500 in PBS, Calbiochem, 481001, EMD Millipore, MA, USA). After blocking with 1% BSA for 1 hr, a secondary anti-human neutrophil elastase antibody was applied (1:2000 in PBS, IgG1, cat #: MA1-10608, ThermoScientific, Hudson, NH) followed by the addition of a horse radish peroxidase-linked (donkey) anti-mouse IgG antibody (1:2000 in PBS, NA934V, GE Healthcare, UK) for 1 hr at room temperature. Blue coloration developed in the presence of the Pierce TMB Substrate Kit (ThermoFisher Scientific, Waltham, MA, USA), and results were quantitated using a human neutrophil elastase standard.

### *P*. *aeruginosa* flagellin ELISA

Flagellin concentrations in *P*. *aerugin*osa lysates were quantitated by ELISA established in this manuscript. Bacterial cultures were sonicated after repeated washes and their density was adjusted as described above. Bacterial lysates were centrifuged twice (10,000 g, 15 min), and supernatants were used in the ELISA. Supernatants of bacterial lysates and *P*. *aeruginosa* flagellin standards (Invivogen, San Diego, CA) were immobilized to 96-well high-binding capacity ELISA plates (Greiner Bio-one, Frickenhausen, Germany) by mixing them with an equal volume of 100 mM carbonate/bicarbonate buffer and incubating the samples overnight at 4°C. Plates were washed three times with PBS containing 0.1% Tween-20 (Sigma, St. Louis, MO, USA) and blocked by 5% bovine serum albumin (Hyclone, Logan, Utah) for 3 hrs at room temperature. After three washes with Tween-20/PBS, anti-*P*.*aeruginosa* flagellin antibody was added (1:250 dilution in PBS, 250 ng/ml, mouse IgG1, hybridoma clone: 18D7, Invivogen, San Diego, CA) and incubated overnight at 4°C. Samples were washed again three times, followed by addition of a secondary horse radish peroxidase-labelled, sheep anti-mouse IgG antibody (1:1000 dilution in PBS, GE Healthcare Bio-Sciences, Pittsburgh, PA, USA) for 30 min (room temperature, dark). After four repeated washes with PBS/Tween-20, color reaction was developed with 3,3’,5,5’-tetremthylbenzidine (TMB, 0.16 mg/mL, Sigma, St. Louis, MO) peroxidase solution and the reaction was stopped by adding 1M HCl. Absorbance was read at 450 nm with either Eon (BioTek, Winooski, VT) or Varioskan Flash (ThermoScientific, Hudson, NH) microplate photometers. Absolute quantitation of *P*. *aeruginosa* flagellin concentrations were calculated using the standard curve and expressed as micrograms per milliliter (μg/ml) or “ng/6x10^8^ bacteria.”

### NET quantitation: MPO-DNA and HNE-DNA ELISA

NETs (MPO-DNA and HNE-DNA complexes) in human PMN supernatants were quantitated by specific ELISA assays as described [41, 53]. Briefly, supernatants of attached human PMNs were treated with 1 μg/ml DNAseI to achieve limited DNA digestion [41, 53]. Diluted samples were added to and incubated overnight on ELISA plates pre-coated with anti-MPO or anti-HNE capture antibodies, followed by addition of horseradish peroxidase-labelled anti-DNA detection antibody [41, 53]. Coloration of added TMB substrate solution (Thermo Scientific, Hudson, NH) was stopped by 1N HCl and absorbance (450 nm) was read either with Eon (BioTek, Winooski, VT) or Varioskan Flash (ThermoScientific, Hudson, NH) microplate photometers. “NET concentrations” were expressed as percentage of the “NET-standard,” consisting of pooled supernatants (5 donors) of PMA-stimulated human PMNs after limited DNAseI-digestion, and were handled parallel with unknown samples [53].

### Phagocytosis

Phagocytosis of PAO1 strains by human PMNs was assessed by measuring the decrease in the number of extracellular (non-phagocytosed) bacteria over time. Human PMNs were mixed with 10 MOI of PAO1 WT or *fliC* and incubated for 60 min. At different time points (0, 2, 20, 40 and 60 min), aliquots were taken, added to ice-cold PBS and centrifuged (300 g, 3 min, 4°C) to pellet PMNs but leave extracellular bacteria in the supernatant. The centrifugation step was repeated once. 100 μL volume of the supernatant was added to 900 LB growth medium, and bacterial concentration was determined using a microplate-based assay [102].

### Superoxide production

Superoxide production was measured by two different assays: Lucigenin-based or Diogenes-based superoxide chemiluminescence kits (National Diagnostics, Atlanta, GA) [19, 29, 40, 41]. 100,000 PMNs adhered to 96-well white plates for 15 min at 37°C in HBSS containing 1% serum. Cells were stimulated by *Pseudomonas aeruginosa* strains (10 MOI), PMA (100 nM) or left unstimulated. Chemiluminescence was measured by a Varioskan Flash microplate luminometer (Thermo Scientific, Waltham, MO, USA) for 90 min. Data are shown as kinetics of representative curves (relative luminescence units, RLU) or integral superoxide production by analyzing accumulated luminescence for the entire duration of the measurement.

### Measurement of peroxidase activity

Myeloperoxidase activity was measured by hydrogen peroxide-dependent oxidation of Amplex Red as described [19]. Undiluted neutrophil supernatants were mixed with assay solution containing 100 μM Amplex Red (Sigma, St. Louis, MO) and 100 μM hydrogen peroxide (Sigma, St. Louis, MO). Production of the fluorescent product was measured in 96-well black plates using a fluorescence microplate reader (Varioskan Flash, ThermoScientific, Waltham, MO, USA) for 30 min at 560 nm excitation and 590 nm emission wavelengths. Calibration was achieved using an MPO standard [19].

### Immunoblot analysis

*P*. *aeruginosa* PAK strains were grown overnight in LB liquid medium, washed and resuspended in RIPA Lysis and Extraction buffer (ThermoFisher Scientific, Waltham, MA USA) before sonication. Bacterial lysates were collected as supernatants after centrifugation (14,000 g, 20 min, 4°C). Protein concentrations were determined with a Pierce BCA Protein Assay Kit (ThermoFisher Scientific, Waltham, MA USA). Equal amounts of proteins from bacterial lysates were loaded onto Novex 8–16% Tris-Glycine Gel together with molecular weight standards and run for 120 min at 110 V. Samples were blotted onto nitrocellulose membrane using the iBlot dry blotting system (Life Technologies, Carlsbad, CA). Membranes were blocked in 5% milk for 1 hr and probed with the primary antibody (anti-*P*. *aeruginosa* flagellin antibody, 1:250, 250 ng/ml, mouse IgG1, hybridoma clone: 18D7, Invivogen, San Diego, CA) overnight at 4°C. After three washes, the secondary antibody was added (HRP-labelled goat anti-mouse IgG, 1:2000, ThermoFisher Scientific, Waltham, MA, USA) for 1 hr at room temperature. Following three repeated washes, blots were probed with the Amersham ECL Western Blotting Detection Kit (GE Healthcare Life Sciences, Pittsburgh, PA, USA), and chemiluminescence was recorded with Konica Minolta SRX-101A developer using HyBlot CL Autoradiography films (Denville Scientific, Holliston, MA).

### Statistical analyses

Results were analyzed by Student's *t*-test or one-way ANOVA. Each experiment was independently performed at least three times with PMNs isolated from different donors. Statistically significant differences were considered as *, p<0.05; **, p<0.01; ***, p<0.001.

## Supporting Information

S1 Fig Characterization of flagellum-deficient *P*. *aeruginosa* strains. (TIF)

S2 Fig Description of the *P*. *aeruginosa* flagellin ELISA. (TIF)

S3 Fig The HNE-DNA ELISA does not detect HNE, MPO, DNA or human chromatin alone. (TIF)

S4 Fig *Shigella flexneri* flagellin does not trigger NET release in human neutrophils. (TIF)

S5 Fig Characterization of bone marrow-derived murine neutrophils. (TIF)

S6 Fig Heat treatment impairs *P*. *aeruginosa*-stimulated ROS production and NET formation in human neutrophils. (TIF)

S7 Fig Superoxide production in human neutrophils stimulated with *motCD*-deficient *P*. *aeruginosa* and its complemented strain. (TIF)
