# Supplementary figures and images for: Swimming Motility Mediates the Formation of Neutrophil Extracellular Traps Induced by Flagellated Pseudomonas aeruginosa
Source: PLoS Pathog. 2016 Nov 17;12(11):e1005987. doi: 10.1371/journal.ppat.1005987 (PMC5113990; doi:10.1371/journal.ppat.1005987)

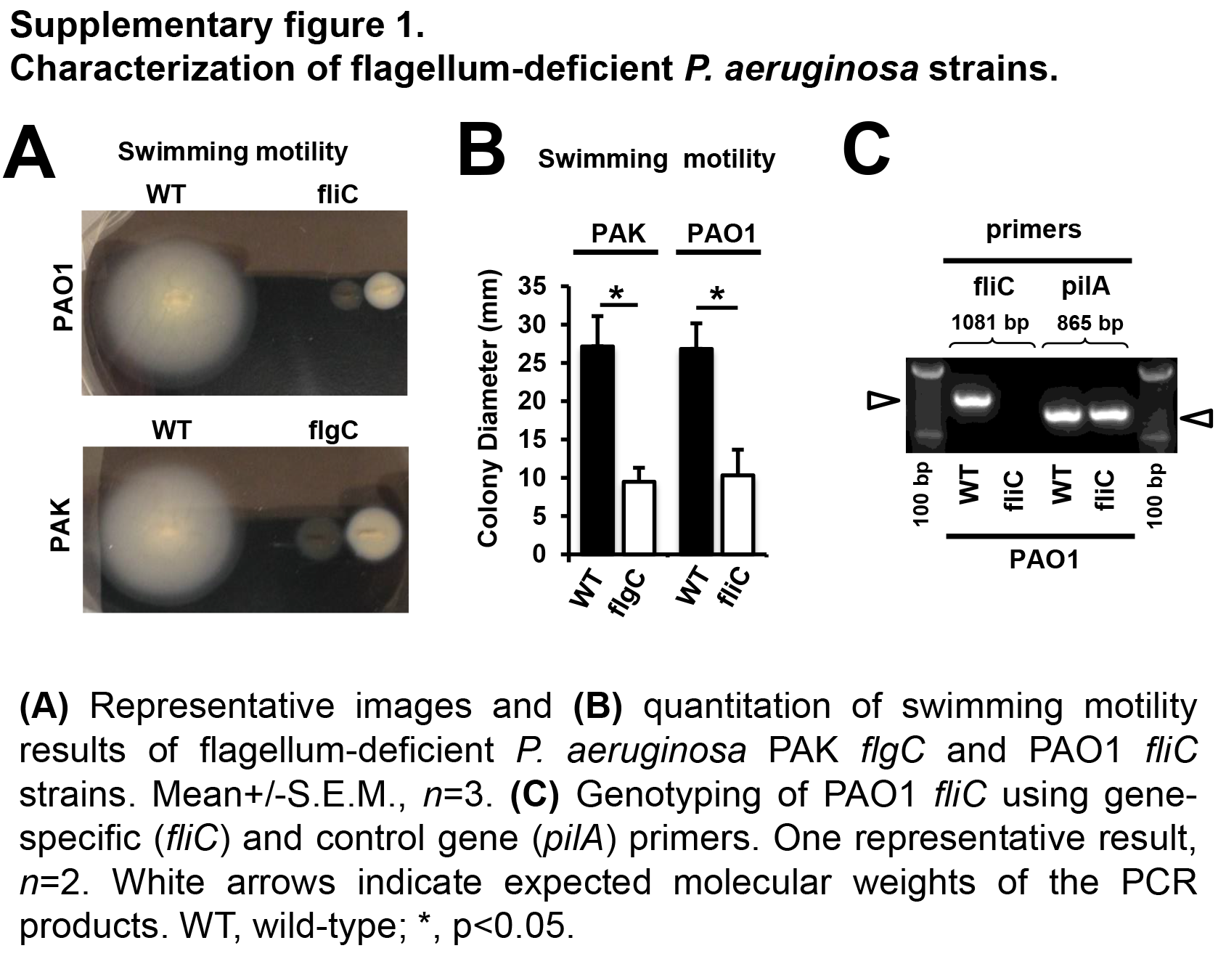

Supplement: S1 Fig — (TIF) [file ppat.1005987.s001.tif]

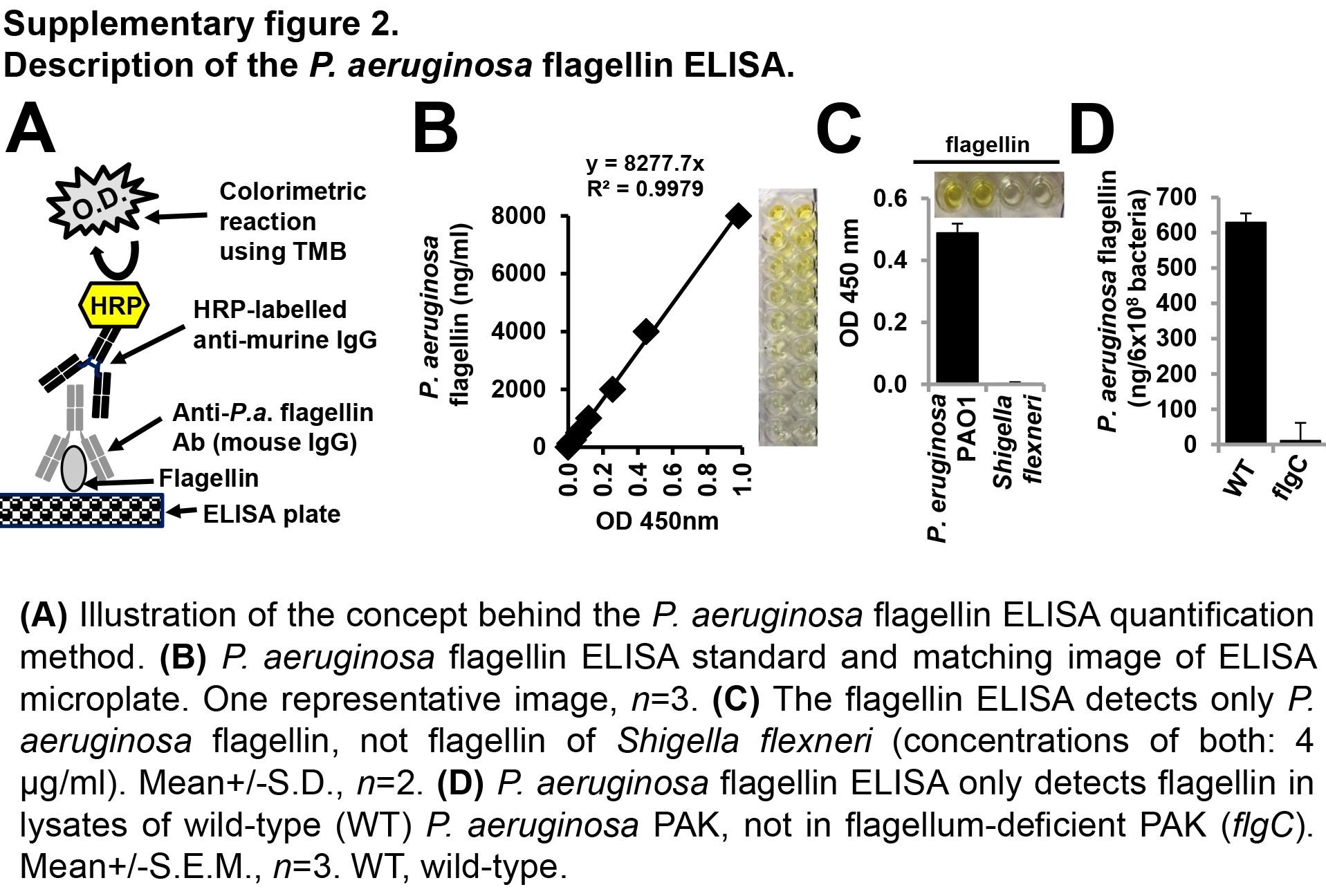

Supplement: S2 Fig — (TIF) [file ppat.1005987.s002.tif]

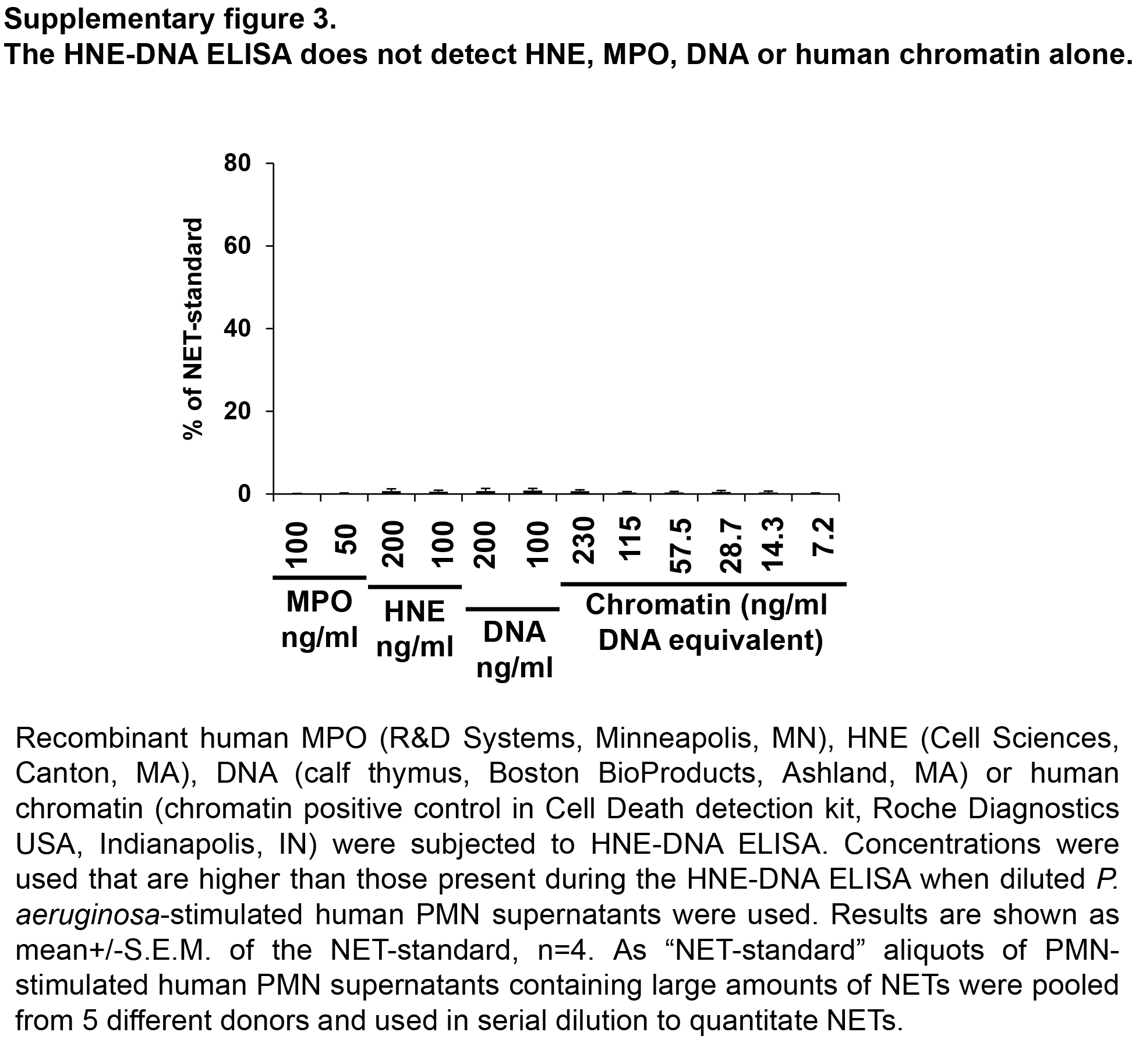

Supplement: S3 Fig — (TIF) [file ppat.1005987.s003.tif]

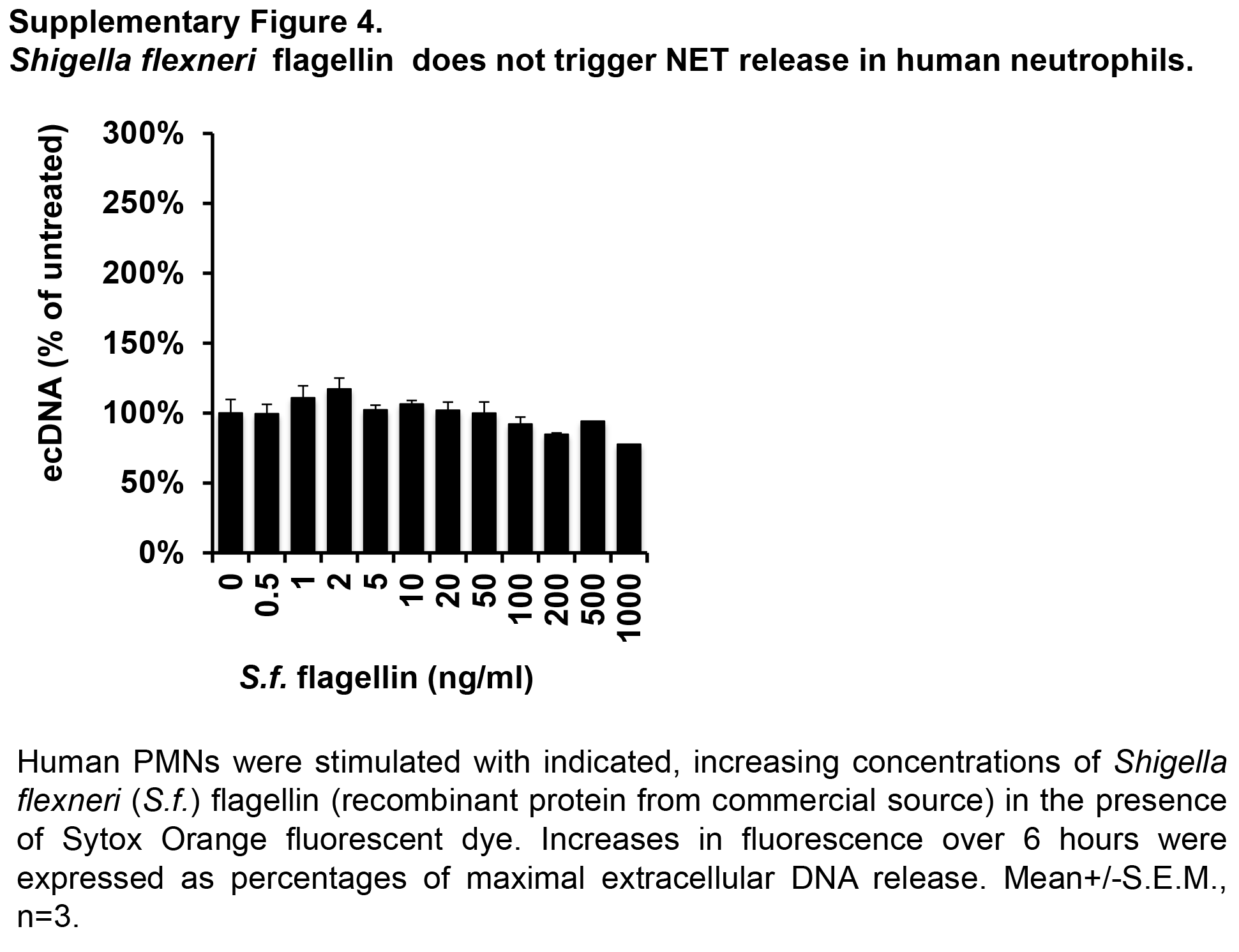

Supplement: S4 Fig — (TIF) [file ppat.1005987.s004.tif]

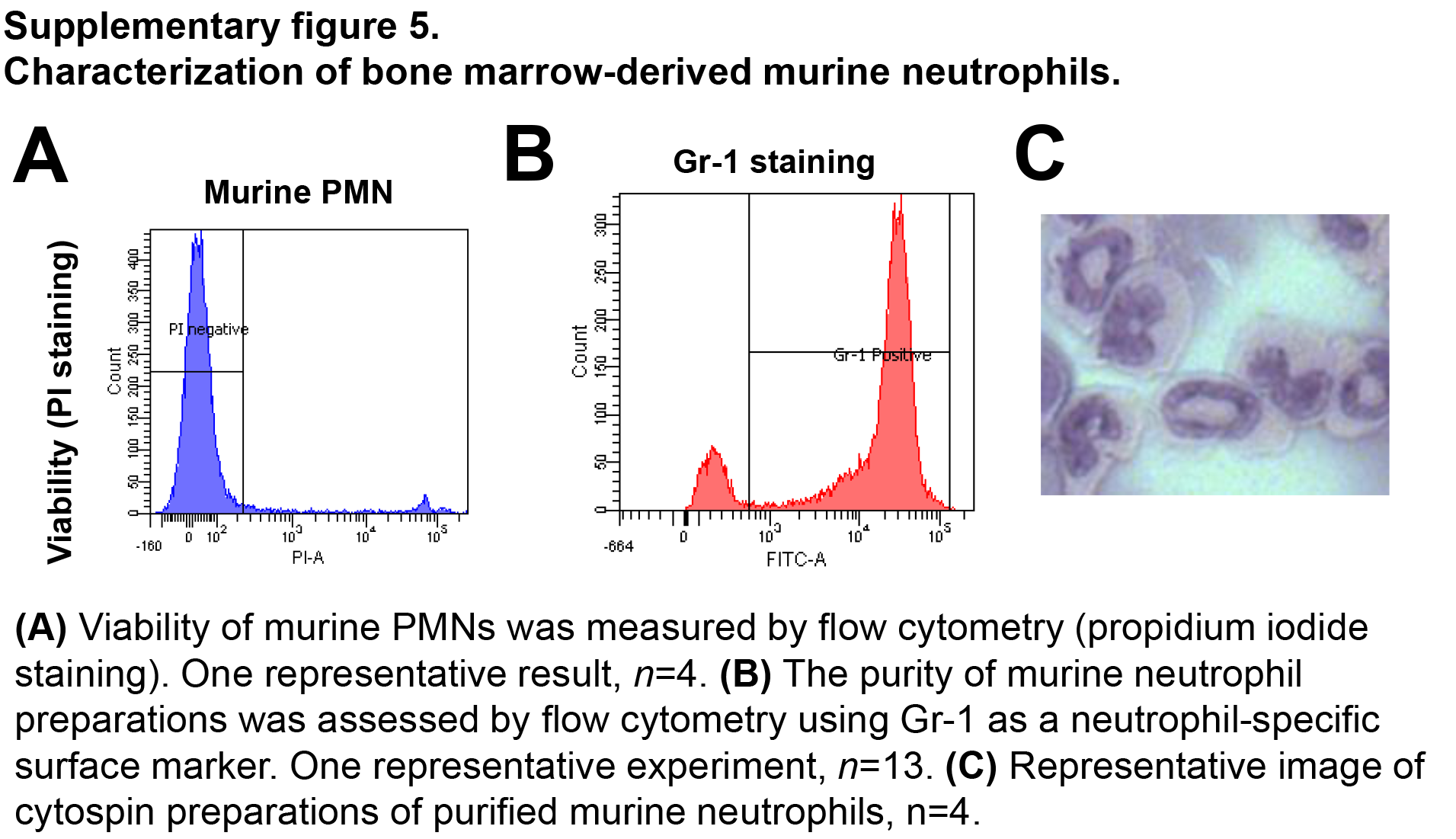

Supplement: S5 Fig — (TIF) [file ppat.1005987.s005.tif]

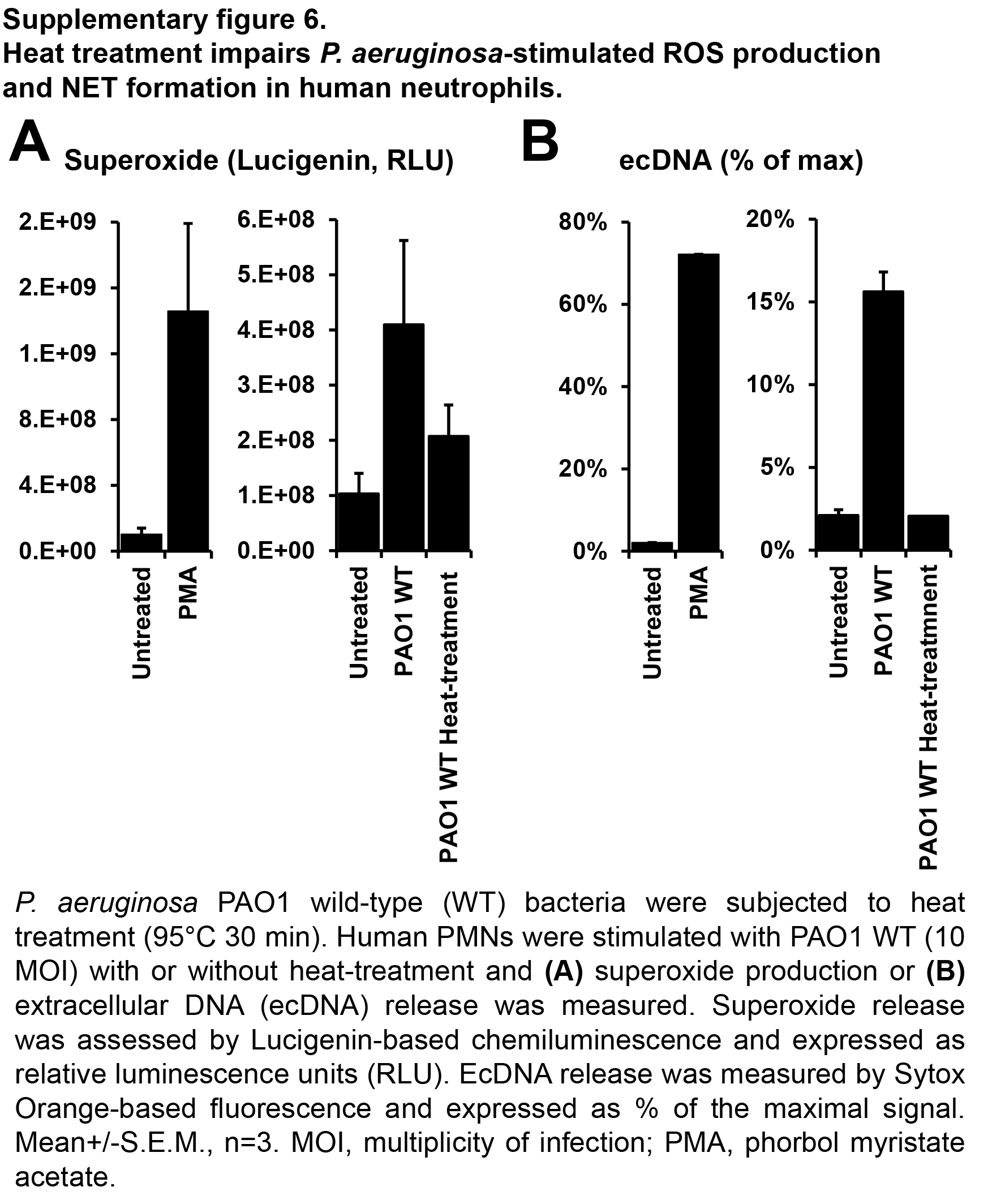

Supplement: S6 Fig — (TIF) [file ppat.1005987.s006.tif]

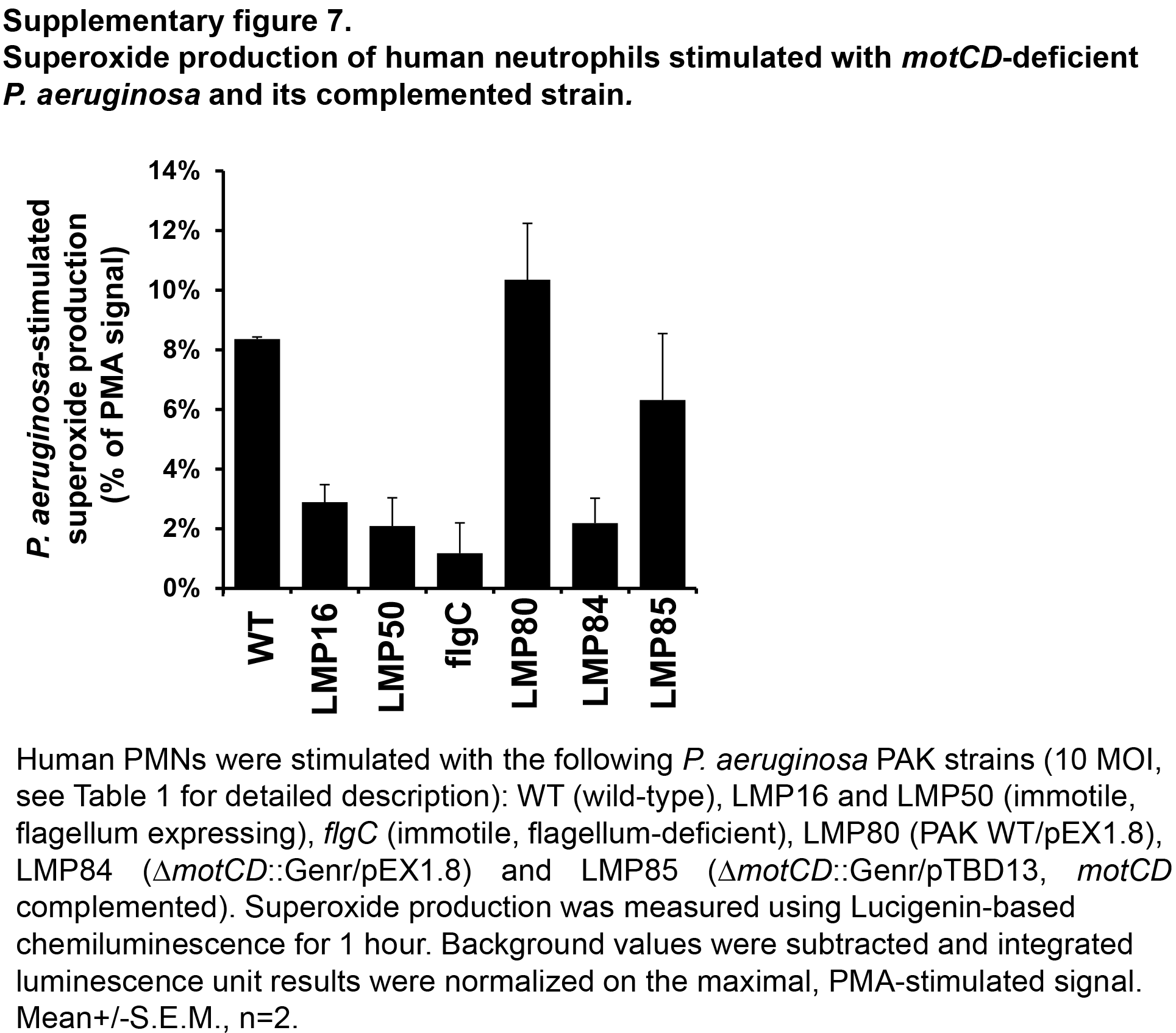

Supplement: S7 Fig — (TIF) [file ppat.1005987.s007.tif]
